# Supplementary material for: An empirical model of aminoacylation kinetics for E. coli class I and II aminoacyl tRNA synthetases
Source: PLoS Comput Biol. 2025 Aug 12;21(8):e1013353. doi: 10.1371/journal.pcbi.1013353 (PMC12367143; doi:10.1371/journal.pcbi.1013353)
Supplement: S1 Text — (PDF) [file pcbi.1013353.s002.pdf]

S1 Text

1. Variation of Kinetic Parameters for Class I and II Enzyme Models

In this section, a systematic variation of the kinetic parameters is performed in order to identify the subset which have the largest effect on k_{cat} and K_m values. This is done for both pyrophosphate exchange kinetics and for the overall aminoacylation reaction. The effects of kinetic parameters on the overall chemistry rate (k_{chem}) and the single turnover rate for aminoacyl transfer (k_{tran}) are also examined. This allows for the identification of the critical parameters which effect all the features of aminoacyl tRNA synthetase kinetics that have been experimentally observed.

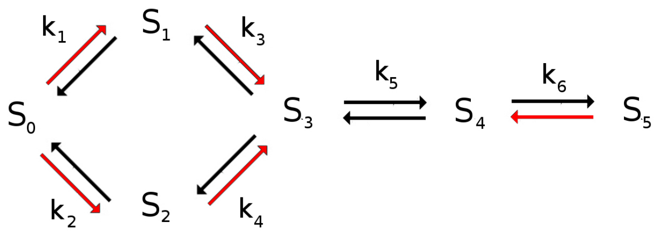

Fig A. Kinetic reaction scheme for the pyrophosphate exchange reaction. State S_0 represents the AARS enzyme, while S_1 and S_2 are the amino acid and ATP bound enzymes, respectively. State S_3 is the enzyme with both amino acid and ATP bound, S_4 is the adenylate and pyrophosphate bound state, while state S_5 is the adenylate bound enzyme state. States follow that of Fig 1 in the main text, with red arrows indicating substrate association to the enzyme.

Effect of Varying Kinetic Parameters on Pyrophosphate Exchange. Fig A gives the reversible reaction network that is used to simulate pyrophosphate exchange. Although there are a total of 12 parameters that can be varied, the 12 dimensional search space can be greatly simplified to a smaller region which reproduces the experimentally observed k_{cat} and K_m values. Specifically, I will reduce the search space to a 4-dim space encompassing the dissociation constants for amino acid and ATP, K_{d1}, K_{d2}, and the amino acid activation rates k_{5f} and k_{5b}. Although there are probably other regions of the parameter space which will reproduce the experimentally observed k_{cat} and K_m's, this region of parameter space is consistent with kinetic rate constants that have been explicitly measured for some tRNA synthetases (see S1 spreadsheet and citations within). Thus, this smaller region of parameter space should be sufficient for the construction of empirical kinetic aminoacyl tRNA synthetase models.

Examining Fig A, it is clear that potential parameters that will likely effect the K_m values associated with pyrophosphate exchange are the dissociation constants for amino acid binding ($K_{d1} = k_{1b}/k_{1f}$ and K_{d4}) and ATP binding ($K_{d2} = k_{2b}/k_{2f}$ and K_{d3}). Similarly, k_5 and k_6 are likely to effect k_{cat} values. As a first examination of the parameter space, I set $K_{d1} = K_{d4}$ and $K_{d2} = K_{d3}$ while varying K_{d1} and K_{d2} between 10 and 1000 μM. In addition I fix $k_{5f} = 180s^{-1}$ and $k_{5b} = 400s^{-1}$ with $k_{6f} = 5\ \mu M^{-1}s^{-1}$ and $k_{6b} = 3000s^{-1}$. The results of this variation are shown as contour plots in Fig B (a-c). As expected, varying the K_d for ATP or amino acid altered the corresponding K_m value and suggests that for pyrophosphate exchange, $K_d \approx K_m$. Varying of the forward rates (k_{1f} and/or k_{2f}) had negligible effect on either k_{cat} or K_m establishing that the predominate effect on the K_m values in the pyrophosphate exchange reaction is due to the overall K_d of amino acid or ATP.

Next, I fix $K_{d1} = K_{d4} = 54\ \mu$M and $K_{d2} = K_{d3} = 600\ \mu$M along with $k_{6f} = 5\ \mu\ \text{M}^{-1}s^{-1}$ and $k_{6b} = 3000s^{-1}$, and systematically vary k_{5f} and k_{5b} between values of 10 and 400 s^{-1}. The contour plots in Supplementary Figure B (d-f) show the effects on k_{cat} and K_m values in the pyrophosphate exchange reaction. As can be seen, K_m increases toward the K_d value of the substrate as k_{5f} becomes substantially less then k_{5b}. In contrast, the overall k_{cat} value increases as k_{5f} increases.

Finally, I examine varying k_{6f} from 0.1 to 200 $\mu\text{M}^{-1}s^{-1}$ and k_{6b} between 10 and 2000 s^{-1}. The values of $K_{d1} = K_{d4} = 54$ μM and $K_{d2} = K_{d3} = 600\ \mu$M are fixed along with $k_{5f} = 180s^{-1}$ and $k_{5b} = 400s^{-1}$. As can been seen from the contour plots in Fig B (g-i), there is little effect on K_m values for ATP or the amino acid, while k_{cat} values start to plateau when $k_{6b} > 1000s^{-1}$. Moreover, there is limited effect on k_{cat} via variation of k_{6f}. Thus, k_{6f} can be chosen to match the measured pyrophosphate dissociation constant which is in the range of $320 - 500\mu$M (1, 2). All together this demonstrates that, by altering the K_d's for the amino acid and ATP, along with k_{5f} and k_{5b}, one can obtain k_{cat} values for pyrophosphate exchange in the range of $20 - 100\ s^{-1}$ and K_ms between 0 and the K_d of the substrate.

Effect of Varying Kinetic Parameters on Aminoacylation. Fig C gives the reversible reaction network that is used to simulate the overall aminoacylation reaction of an aminoacyl tRNA synthetase for class I enzymes. Here there are a total of 44 parameters that can be varied to potentially effect overall aminoacylation kinetics. However, some of these rates, in particular the binding of tRNA and aa-tRNA to the enzyme (rates k_0^x with $x = a, b, c, d, e$ and k_{11}, respectively) and the transfer rate (k_{8f}), have usually been experimentally measured. Moreover, if one first adjusts the 12 parameters k_1 to k_6 to fit pyrophosphate exchange and assumes that these rates in the presence of tRNA are the same, i.e. $k_1 = k_1^t$ etc., then this leaves just k_7, k_9 and k_{10} to vary.

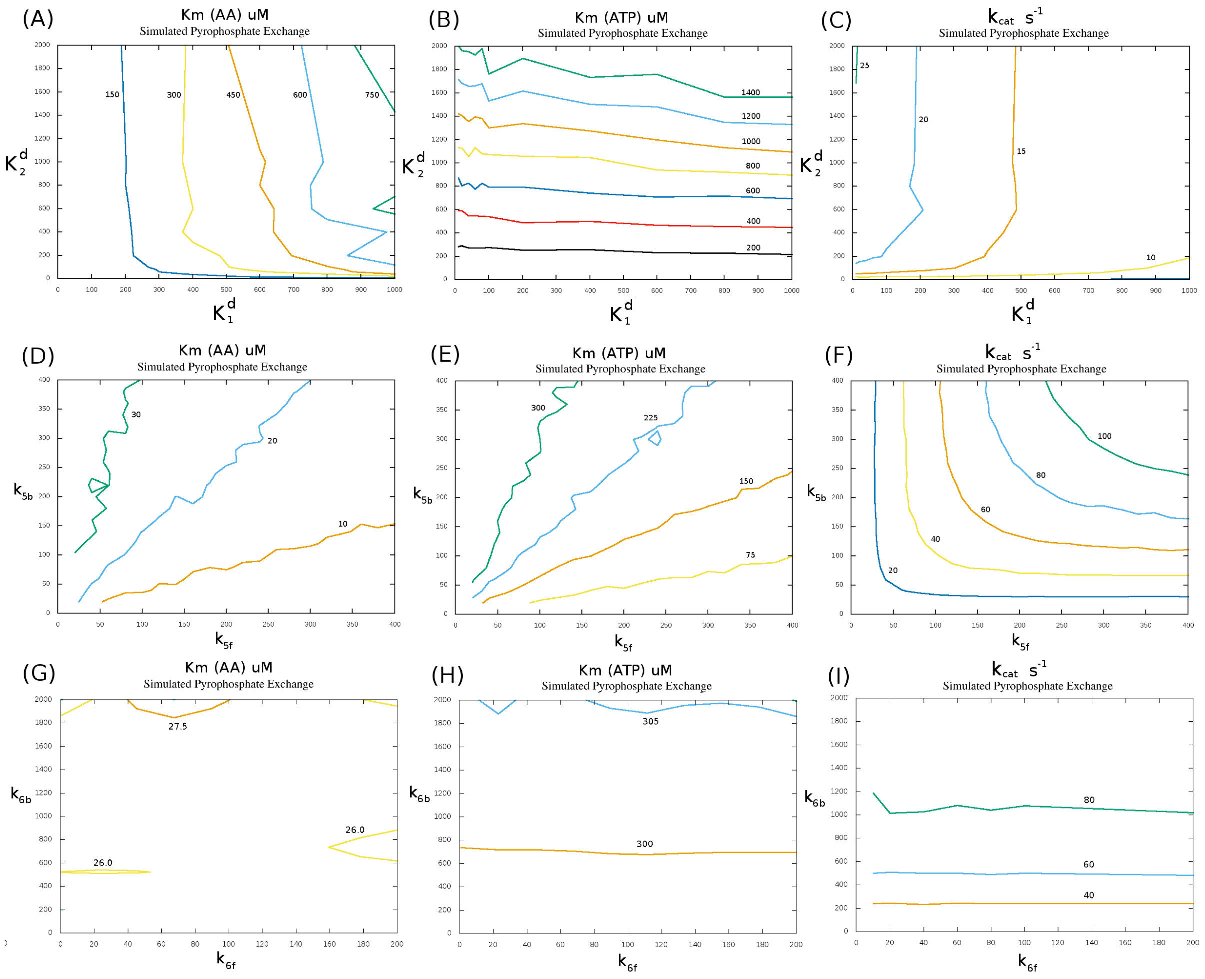

Fig B. Effect of parameter variation on pyrophosphate exchange kinetics. (A-C) Contour plots of the effect on Michaelis–Menten constant K_m and pyrophosphate k_{cat} as the amino acid and ATP dissociation constants are varied. (D-F) Contour plots of the effect on the Michaelis–Menten constant K_m and pyrophosphate k_{cat} as the amino acid activation rates k_{5f} and k_{5b} are varied. (G-I) Contour plots of the effect on the Michaelis–Menten constant K_m and pyrophosphate k_{cat} as the pyrophosphate release rates k_{6f} and k_{6b} are varied.

The rate k_{10} governs the reversible binding of AMP to the enzyme after amino acid transfer to the tRNA. This dissociation rate has been estimated based on experimental observations to have a range of $K_d = k_{10b}/k_{10f} \approx 1200 - 2400\mu$M (2, 3). Variation of this parameter has negligible effect on K_m or k_{cat} (obviously as long as AMP release is greater than the overall catalysis rate - $k_{10b} > k_{cat}$) but alters the overall steady-state percentage of charged tRNAs in the presence of different concentrations of AMP. Values of this parameter will be important for insuring tRNAs charge in the presence of cellular concentrations of free AMP. Total AMP concentrations in *E. coli* have been estimated at 180-250 μM (4), with free AMP concentrations lower then this. Since this parameter has negligible effect on K_m and k_{cat}, I set $k_{10f} = 1\ \mu M^{-1}s^{-1}$ and $K_{10b} = 2400\ s^{-1}$ to insure AMP dissociation does not inhibit the overall aminoacylation rate.

This leaves just k_7 and k_9 to vary in order to attempt to fit the k_{cat} and K_m values for aminoacylation. I relax this condition and additionally allow k_6^t, i.e. pyrophosphate release in the presence of uncharged tRNA, to also vary. Thus, the 38 dimensional space has been potentially limited to a much smaller 6 dimensional space for fitting to experimental k_{cat} and K_m values. The two reaction routes through aminoacylation of tRNA thus comprise, (A) a route where PP$_i$ is first released prior to the transfer of amino acid to the tRNA (governed by rate k_6^t) and (B) a route where PP$_i$ is not released until after transfer of the amino acid to the tRNA (governed by rates k_7 and k_9). I have left both of these routes in for two reasons. First, Kern and Lapointe (5) noted that, for some tRNA synthetases, presence of PP$_i$ may result in conformational changes to the enzyme which bring tRNA closer to the reaction center where the adenylate is present. Second, when I set $k_{7f} = k_{7b} = 0$, forcing PP$_i$ release prior to amino acid transfer, I have found that it is very difficult to reproduce both k_{chem} and K_m values that have been measured experimentally for some tRNA synthetase enzymes. Essentially, a slow release of PP$_i$ is (in general) required to move K_m value closer to the K_d value for the substrate. This has the added problem that it forces PP$_i$ release to become the rate limiting step. For enzymes such as cysRS which display burst kinetics, this will result in the disappearance of the burst kinetics in the model. Pope *et al.* noticed a similar issue when they attempted to theoretically simulate ileRS aminoacylation for *S. aureus* based on an extensive set of experimentally measured kinetic rates for the enzyme (6). In this work they noticed that, unless PP$_i$

was modelled as being released *after* transfer of the amino acid to the tRNA, K_m values for ATP and isoleucine where far below what was measured experimentally. Thus, for all these reasons, I have included a reaction path which allows amino acid transfer in the presence of pyrophosphate.

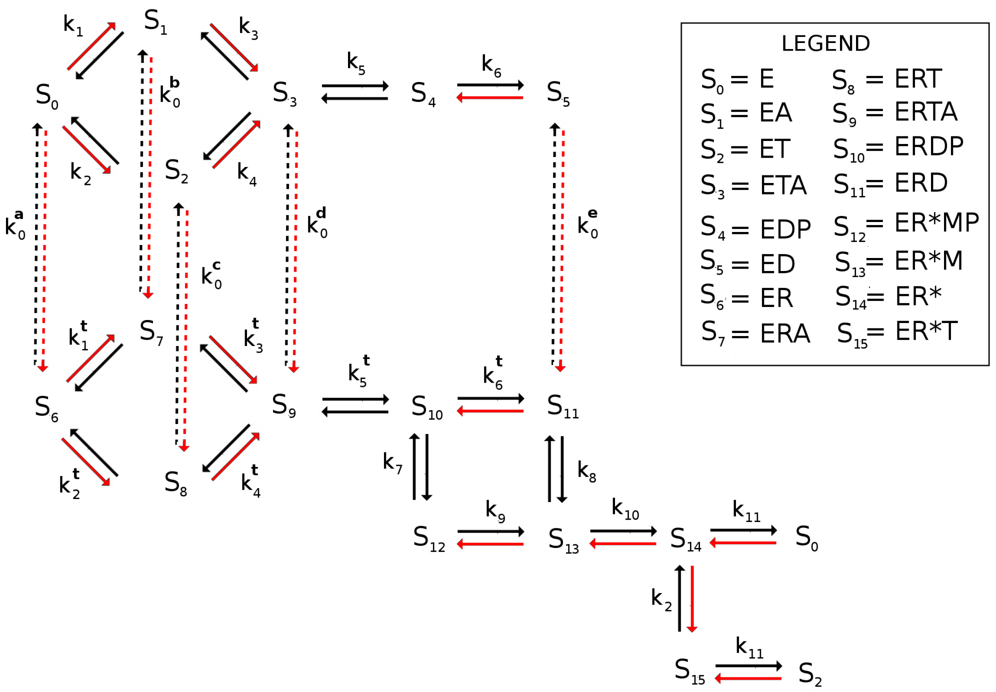

Fig C. Kinetic reaction scheme for Class I aminoacyl tRNA synthetases. Reaction scheme diagram labelling the individual kinetic reactions in the aminoacylation reaction for the class I aminoacyl tRNA synthetase enzymes. Individual kinetic rates for each reaction can be found in Table B. The states of the enzyme S_i are given in the legend on the right with abbreviations E = AARS enzyme, T = ATP, M = AMP, P = PP$_i$, D = AA Adenylate, R = tRNA, and R* = AA-tRNA denoting the charged tRNA. For example, state S_0 corresponds to the enzyme free of all substrates while S_5 corresponds to the enzyme with bound amino acid adenylate.

For the class II reaction scheme shown in Fig D, the binding of substrates can occur at two different sites. I model the tRNA charging kinetics based on the flip-flop mechanism detailed by Guth et al. (7). This mechanism is based on experimental observations that tRNA charging occurs first in site 1, followed by activation of the amino acid and formation of the adenylate before the charged tRNA in site 1 is released. I model the charged tRNA as being able to leave site 1 as soon as the adenylate is formed in site 2. The mechanism and rates follow similar fitting strategy to that of class I enzymes, as one first uses any experimental data on single turnover kinetics and pyrophosphate exchange to fit the kinetic parameters for site 1 of the enzyme. The remaining kinetic rates for site 2 can be varied straightforwardly following a similar scheme to that of pyrophosphate exchange to fit the model to the experimental data for K_m and k_{cat} of aminoacylation.

2. Kinetic Parameters for Aminoacyl tRNA Synthetases

Following the procedure outlined in the previous section, I have identified a set of kinetic parameters which reproduce the experimentally observed k_{cat} and K_m values for each of the 20 aminoacyl tRNA synthetases. For most of the enzymes, there are multiple measurements of both k_{cat} and K_m for the pyrophosphate exchange and aminoacylation reactions from several different experimental groups (see S1 Spreadsheet). In these cases, after removing outliers, I have used the average of the k_{cat} and K_m data to fit the empirical models to. It is important to note, that some of the fitted k_{cat} values for the aminoacylation reaction are above the average of the measurements, and occasionally outside the range of values found experimentally. This is to insure that the overall turnover rate supported by the AARS enzyme is sufficiently fast to support the turnover rate needed for exponential growth in *E. coli.* Table B and Tables C/D give the kinetic rates for class I and class II aminoacyl tRNA synthetases, respectively, which fit the experimental measurements reported in S1 Spreadsheet. Information on the values of k_{cat} and K_m that the empirical model produces for each of the enzymes can also be found in Table I.

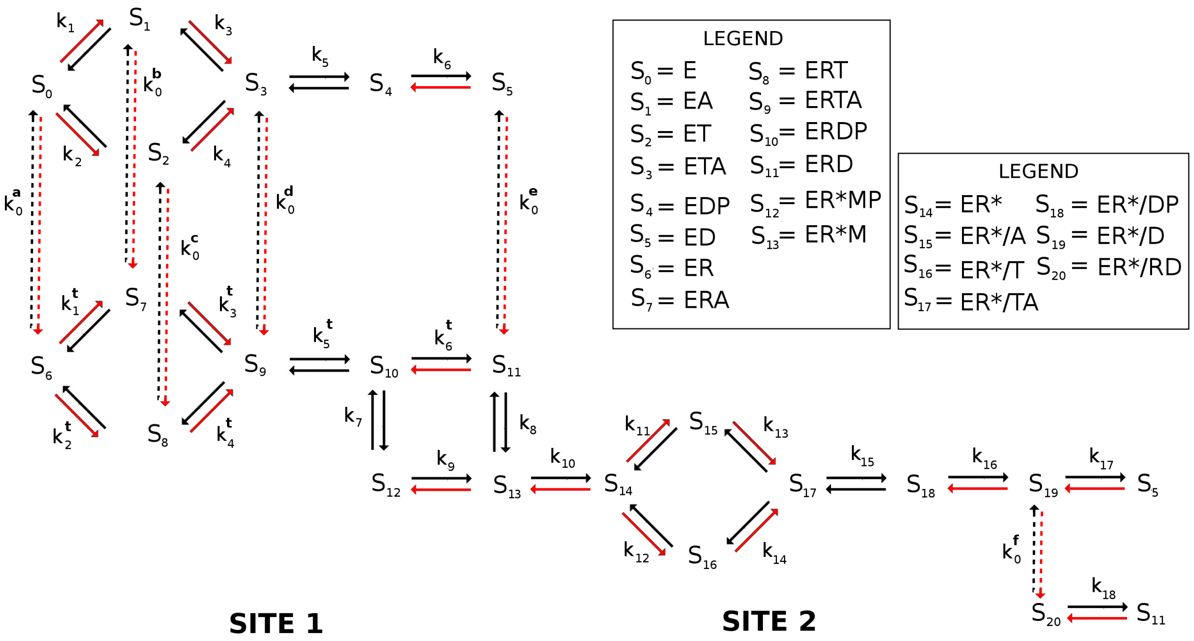

Fig D. Kinetic reaction scheme for Class II aminoacyl tRNA synthetases. Reaction scheme diagram labelling the individual kinetic reactions in the aminoacylation reaction for the class II aminoacyl tRNA synthetase enzymes. Individual kinetic rates for each reaction can be found in Tables C and D. The states of the enzyme S_i are given in the legend on the right with abbreviations E = AARS enzyme, T = ATP, M = AMP, P = PP$_i$, D = AA Adenylate, R = tRNA, and R* = AA-tRNA denoting the charged tRNA. For example, state S_0 corresponds to the enzyme free of all substrates while S_5 corresponds to the enzyme with bound amino acid adenylate. States with substrate bound in the first and second site are separated by a slash, *e.g.* state S_{15} with charged tRNA in the first site and AA is bound to the second catalytic site is denoted as ER*/A.

3. Calculation of *in vivo* Amino Acid Usage and tRNA Turnover Rates

In this section I discuss how the amino acid usage and tRNA turnover rates are computed from computational simulations of ribosome translation that would occur *in vivo*. Additionally, recent proteomics data of AARS numbers in *E. coli* cells growing at different rates are compared with previous 2-D electrophoresis data from Neidhardt *et al.* (8).

Estimation of AARS Numbers from Proteomics Data. Jakubowski and Goldman (9) previously measured amino acid incorporation into protein using ^{3}H labelled amino acids and used measurements of AARS numbers from Neidhardt *et al.* (8) to estimate average tRNA charging rates. Based on the measurements of AARS numbers using 2-D gel electrophoresis data from Neidhardt *et al.* and the estimated tRNA charging rates computed in (9), it has been suggested that the k_{cat} values measured experimentally *in vitro* for most AARSs are too small to support the rates of tRNA charging that are estimated for exponentially growing *E. coli* (10). However, it is important to note that Neidhardt *et al.* stated in their discussion that their results would only reflect the actual numbers of AARS in cell if one accepted that they achieved near 100% efficiency in extraction of AARS enzymes from cell lysates. Thus, it is reasonably possible that the Neidhardt measurements are underestimated along with several of the amino acid incorporation rates measured by Jakubowski and Goldman's (9) being overestimated. This would result in an overall overestimate of the expected tRNA charging rate *in vivo*.

Indeed, examining the data in Table E reveals the amount of AARS enzyme reported in Jakubowski and Goldman (9) is substantially lower then what was measured more recently by Mori *et al.* (11). Moreover, while the Jakubowski and Goldman measurements of amino acid usage for several amino acids (e.g. valine, isoleucine, leucine, tryptophan, proline, histidine and lysine) are very close to theoretical estimates based on the number of translating ribosomes, there are several which are substantially different (glutamine, asparagine). In fact, the Jakubowski and Goldman data suggest that both cystine and tryptophan are more frequently incorporated into *E. coli* proteins then glutamine or asparagine, which is contradictory to the observation that cysteine and tryptophan are the most rare amino acids in *E. coli* proteins. This suggest that some of these measurements may not be completely reliable. Fortunately over the past decade, several experimental groups have measured absolute concentrations of proteins in exponential growing *E. coli* cells in various growth conditions using either liquid chromatography mass spectroscopy or ribosome profiling methods (11–15). These more recent proteomics data provide additional data that can be compared with Neidhardt *et al.* and give the average numbers of AARSs in exponentially growing *E. coli* cells covering four different growth rates. Table F shows a selection of the data used in this study while the complete proteomics data set of 12 measurements can be found in Tab 2 in S1 Spreadsheet.

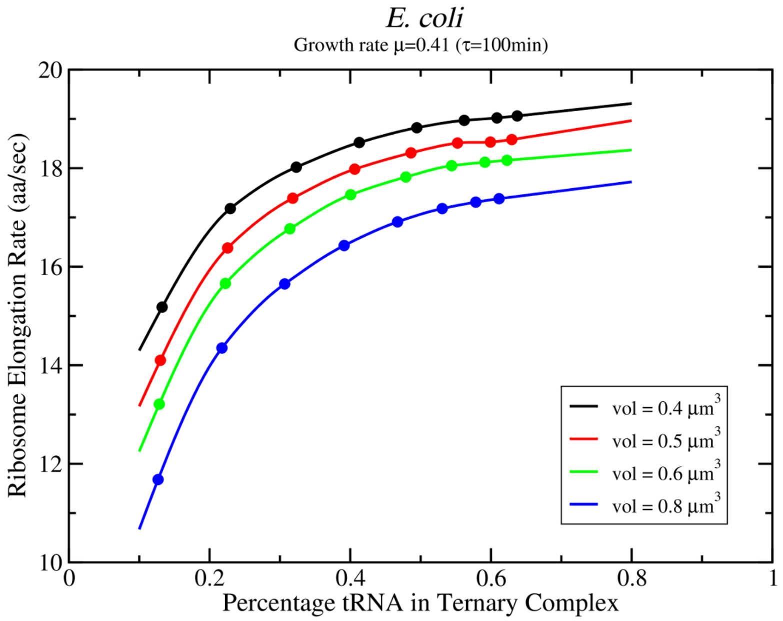

Fig E. The dependence of the translation elongation rate on total ternary complex concentration. Stochastic simulations of translation *in vivo* are used to calculate the average elongation rate of ribosomes in *E. coli* for different cellular volumes verses the percentage of the total amount of tRNA in ternary complex. The fractions of each tRNA isoacceptor in ternary complex set to the optimal values listed in Table G (see section 4 for further details).

Estimation of Amino Acid Usage from Simulations of *in vivo* Translation. One way to reasonably estimate the amino acid usage rate is by taking the total number of elongating ribosomes multiplied by the average elongation rate and the average frequency of an amino acid in *E. coli* proteins. Following standard predictions of Dennis and Bremer (16), the total number of elongating ribosomes can be assumed to be 80% of the total number of ribosomes in the cell which allows for a 5% fraction to be terminating or initiating with the remaining 15% free. Regarding the average elongation rate of ribosomes, this has been estimated by Dennis and Bremer (16) to be 15, 18, and 22 amino acids per second for growth rates of μ =0.41, 0.69, and 1.98 h^{-1}, respectively. However, Dai *et al.* (17) have measured the elongation rates to be slightly slower at 12, 15, and 18 amino acids per second based on translation of LacZ mRNAs. Thus for a given *E. coli* growth rate (*e.g.* 0.41 h^{-1}), one could take the average estimated elongation rate (12 aa/sec) and multiply by the active fraction of ribosomes and the average frequency of amino acids to obtain the amino acid usage rate a_i.

Despite the simplicity of the calculation, there are two issues with this approach. First, the elongation rate and usage rate of individual tRNA isoacceptors is dependent on the tRNA bias in free ternary complex in the cell, as well as the relation of this tRNA bias to the overall codon frequency in the transcriptome (see discussion in section 4 on tRNA bias below). Second, the elongation rate at specific growth rates is further complicated by experiments which observed an increased protein production rate following a nutrient up-shift (18). In these experiments, initially slow growing *E. coli* cells (initial doubling time $\tau_i = 90$ min) had their medium supplemented with glucose and amino acids resulting in an up-shift in protein production and growth rate (final doubling time $\tau_f = 28$ min). Interestingly, the up-shift in protein production occurred within a few minutes (18), faster then would be needed to synthesise more ribosomes, mRNA, tRNA, or AARS enzymes. This suggests that nutrient up-shift causes an increase in the elongation rate and active ribosome fraction to the levels that would be observed in the fastest growing cells, resulting in an overall increase in the protein production rate. These experiments suggest that the number of AARS enzymes in the cell need to be capable of supporting a tRNA charging rate to enable the maximum elongation rate that would occur if a nutrient up-shift took place.

However, what is the maximum elongation rate that is achievable? To answer this question, I have simulated the translation that would occur *in vivo* in *E. coli* cells during exponential growth using my stochastic model of translation (19, 20). This model simulates all known kinetic steps involved in initiation, elongation, and termination in *E. coli* on the full transcriptome of mRNAs that would be present in the cell. For instance, at a growth rate of $\mu = 0.41$, my model simulates the movement of ≈ 8000 ribosomes on a transcriptome of $\approx 10^6$ nucleotides. Moreover my stochastic model does not enforce an exact elongation rate but allows the elongation rate to emerge due to tRNA concentration and competition effects between isoacceptors. Using the optimal tRNA bias (see section 4 below) along with a transcriptome based on mRNA seq data from Li (13), I have calculated the elongation rate as a function of the percentage of the total tRNA that is in ternary complex as shown in Figure E. As can be seen, the elongation rate peaks when roughly 50-60% of the available tRNA is in free ternary complex. At concentrations of ternary complex higher then this, the elongation rate is limited by the time to accommodate the amino acid and translocate the ribosome.

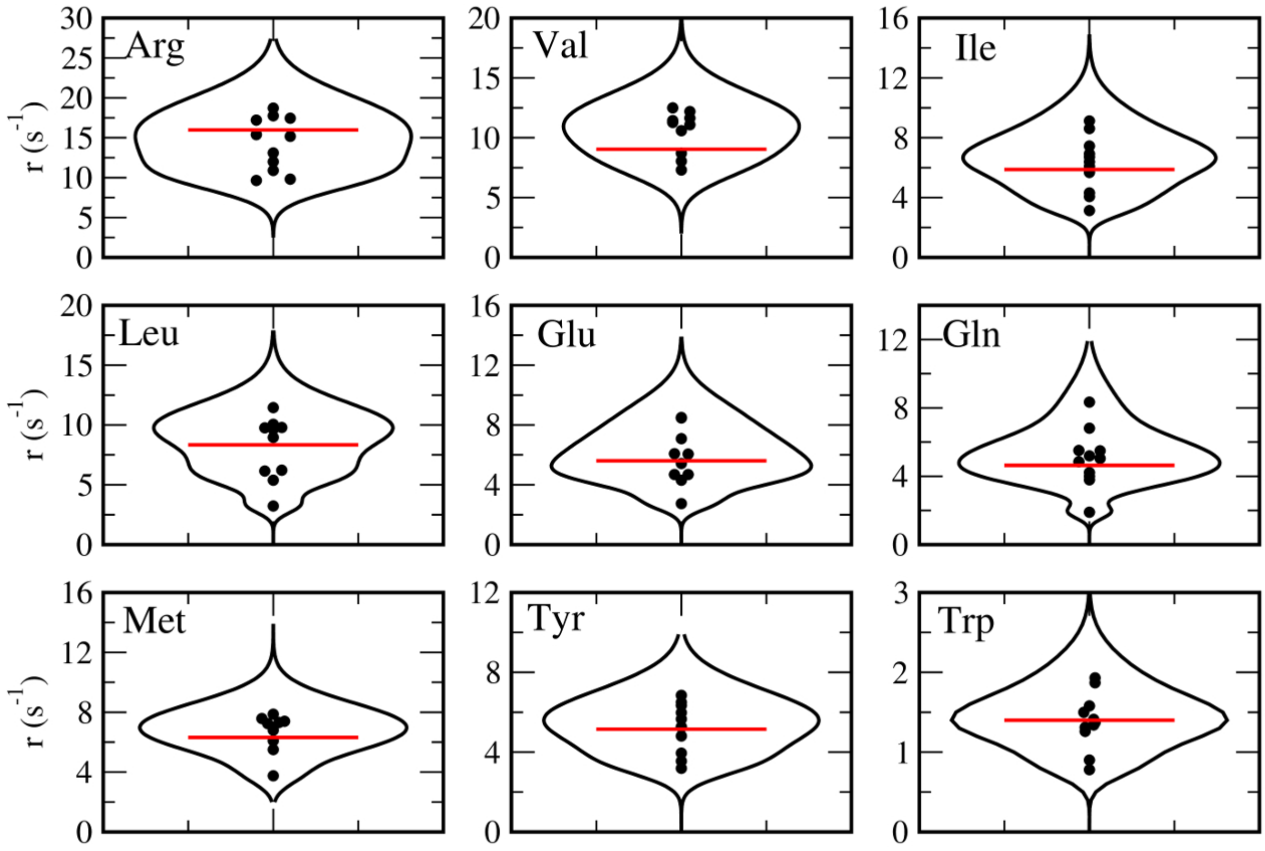

Fig F. Estimated average tRNA turnover rates for class I aminoacyl tRNA synthetases. Violin plots for the AARS tRNA turnover rates are shown for each of the class I enzymes, with cysRS shown in Fig 5 of the main text. Each of the individual proteomics data points used to construct the violin plot are shown (black dots) along with the value used for optimization (red line).

In summary, my stochastic ribosome model along with the following three settings are used to estimate both the usage of amino acids and tRNA isoacceptors; (1) the optimal tRNA fractions are used for the amount of each tRNA in free ternary complex, (2) estimates of the average cellular volumes of $v = 0.45, 0.65, 0.90, 1.95$ μm^3 are used for growth rates of $\mu = 0.41, 0.69, 1.04, 1.73$ (see discussion on cellular volume estimates below) and, (3) half of the available tRNA is forced to reside in free ternary complex. For example, at a growth rate of $\mu = 0.41$ h^{-1}, 37000 tRNAs out of a total of 74000 are forced to reside in free ternary complex (cf. Table F). Amino acid and tRNA isoacceptor usage rates are then calculated based on averages over 30 minutes of translation in a typical *E. coli* cell containing the appropriate number of ribosomes and mRNAs for a given growth rate.

Estimation of AARS Activity *in vivo*. Using the amino acid usage rate estimates from the computer simulations and the number of AARS from proteomics data, I have estimated the tRNA turnover rates (r_i) *in vivo* of the $i = [1, 20]$ aminoacyl tRNA synthetase enzymes from *E. coli*. For each proteomics data point k, the $r_i(k)$ is calculated from

$$r_i(k) = \frac{a_i}{n_i(k)}\ ,$$

where $n_i(k)$ is the number of enzymes and a_i is the amino acid usage calculated from the theoretical translation simulations on the full transcriptome. Table E gives an example of the results for the proteomics data from Mori *et al.* for the *E. coli* growth rate of $\mu = 0.69$ h^{-1} (11).

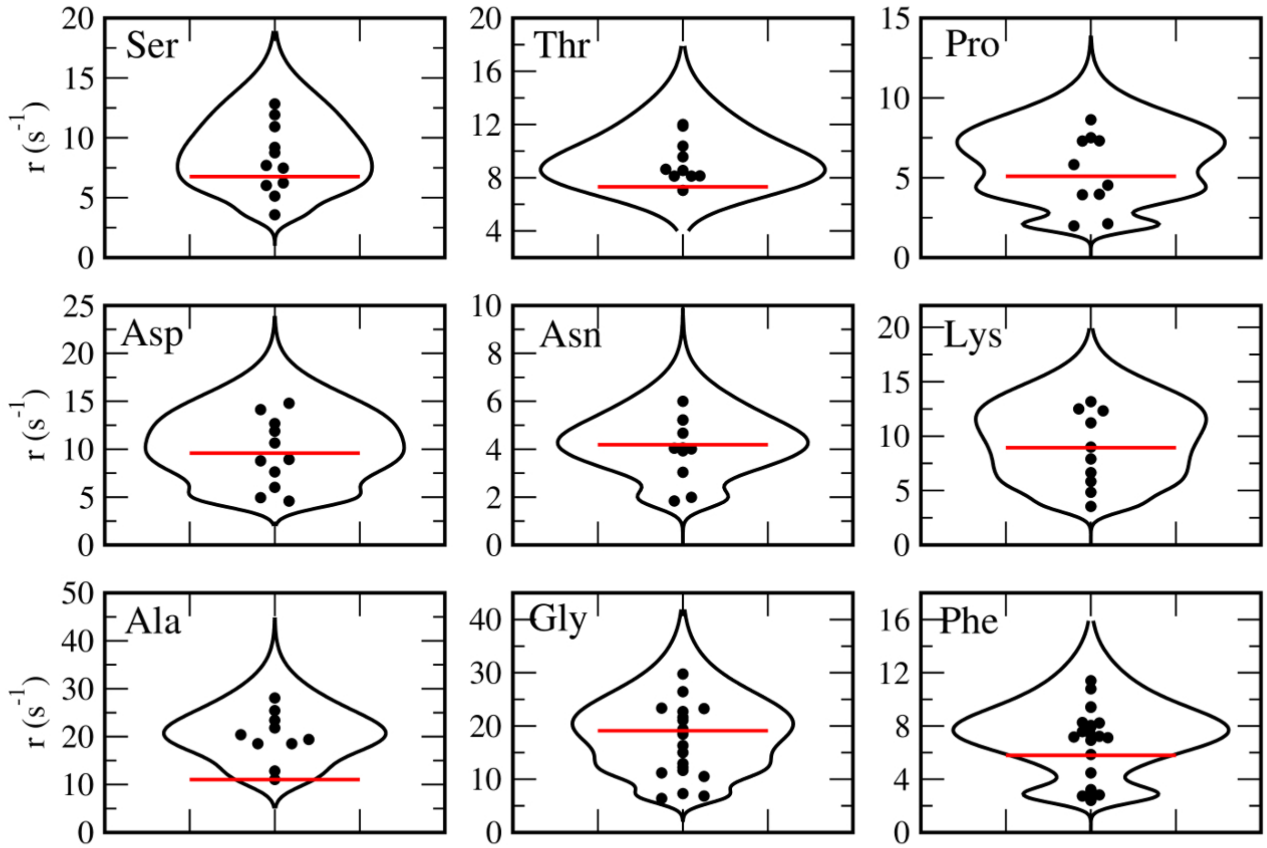

Fig G. Estimated average tRNA turnover rates for class II aminoacyl tRNA synthetases. Violin plots for the AARS tRNA turnover rates are shown for each of the class II enzymes, with hisRS shown in Fig 5 of the main text. Each of the individual proteomics data points used to construct the violin plot are shown (black dots) along with the value used for optimization (red line).

The full set of proteomics data covering 12 measurements was used to construct violin plots shown in Fig F and Fig G along with those in Fig 5 in the main text for cysRS and hisRS. As can be seen in the figures, consensus turnover rates emerge for each of the aminoacyl-tRNA synthetase enzymes, represented by peaks in the violin plots, with a few AARS enzymes (*e.g.* proRS) having more then one potential turnover rate. Red lines in the figures represent the optimised turnover rate that have been used to calculate the average number of enzymes in an *E. coli* cell. Overall there is a good agreement with the consensus turnover rate with the optimised rate that has been used in the simulations with the exception of alaRS. Here alaRS turnover rate was lowered to approximately 10 s^{-1} as single turnover experiments determined $k_{tran} = 16.4$ s^{-1} for *E. coli* alaRS (21) which places an upper limit on k_{cat} since it is expected that $k_{cat} < k_{tran}$.

4. tRNA Bias in Ternary Complex Required For Maximal Ribosome Elongation Rate

In total, *E. coli* has 42 different tRNA isoacceptors which decode the 64 codons of the genetic code. During translation, tRNAs in ternary complex must bind to the ribosome and decode the codon in the A-site of the ribosome. This requires the ribosome to successfully identify the correct tRNA isoacceptor that can decode the current codon in the ribosome A-site from the set of all tRNAs that are present in the cell. Some codons such as CCG, can be decoded by more then one isoacceptor, in this case by either tRNA^{pro1} or tRNA^{pro3}, while other codons have a unique isoacceptor which decodes it. Thus, the time for the correct codon to enter the A-site and decode the codon is directly related to (a) the probability of correctly identifying a tRNA from those present in the cell that is capable of decoding it and (b) the overall concentration of all combined tRNAs. The relative concentrations of the 42 different tRNAs in ternary complex is sometimes referred to as the tRNA bias, and adjusting these relative concentrations will impact on the average decoding time of a given codon due to alteration of the probability that the correct tRNA enters the A-site. Let p_c denote the probability of picking out a correct tRNA from all tRNAs in ternary complex that can decode codon c, while $q_c = (1 - p_c)$ is the probability of picking out an incorrect tRNA. For example, codon CCG can be decoded by either tRNA^{pro1} or tRNA^{pro3}. Thus, p_{ccg} can be calculated as

$$p_{ccg} = \frac{1}{N}(N_{pro1} + N_{pro3}) = n_{pro1} + n_{pro3}\,,$$

where N is the total number of tRNA in ternary complex, N_{pro1} is the total number of tRNA^{pro1} in ternary complex. It is important to note that even though $p_c < 1$ for all codons, the sum

$$\sum_c p_c > 1\,,$$

since tRNA isoacceptors can decode more then one codon causing n_{pro1} (as an example) to appear more then once in the sum. During decoding, selection of the correct tRNA will follow a Bernoulli probability distribution, and each codon will have its own Bernoulli probability p_c. Specifically, there will be k attempts of the *wrong* tRNA binding to the A-site, followed by the $k+1$ th attempt in which the correct tRNA is bound to the ribosome to decode the codon. The total time $\tau(k)$ to identify and bind the correct tRNA where k failures was made is given by

$$\tau(k) = (\tau_{on} + \tau_{off})k + \tau_{on}\,,$$

where τ_{on} is the average time for a tRNA to bind the ribosome and transfer to the A-site for decoding and τ_{off} is the average time for tRNA to unbind from the A-site and be released from the ribosome. Note that, in a first approximation, these times can be considered independent of the codon and tRNA isoacceptor. The average time to identify the correct tRNA to decode codon c (denoted as τ_c) is given as the expectation value of $\tau(k)$ over all possible number of k failures, *i.e.*

$$\begin{aligned} \tau_c = \langle \tau(k) \rangle_c &= \sum_{k=0}^{\infty} q_c^k p_c \tau(k) \\ &= (\tau_{on} + \tau_{off}) \sum_{k=0}^{\infty} k q_c^k p_c + \tau_{on} \sum_{k=0}^{\infty} q_c^k p_c\,. \end{aligned}$$

Using the relations

$$\begin{aligned} \sum_{k=0}^{\infty} q_c^k p_c &= \frac{p_c}{1 - q_c} = 1\,, \\ \sum_{k=0}^{\infty} k q_c^k p_c &= \frac{1}{p_c} - 1\,, \end{aligned}$$

one finds

$$\tau_c = \frac{(\tau_{on} + \tau_{off})}{p_c} - \tau_{off}\,.$$

The average *total* time to decode codon c is the average time to bind the correct tRNA isoacceptor in the A-site (τ_c) plus the average time to accommodate the amino acid and translocate the ribosome to the next codon (τ_a). Thus, one can calculate the average elongation rate in codons per second as $\langle E \rangle = \frac{1}{T}$, where T is the average time to decode any codon, i.e.

$$\begin{aligned} T &= \sum_c \tau_c f_c + \tau_a \\ &= \tau_a - \tau_{off} + (\tau_{on} + \tau_{off}) \sum_c \frac{f_c}{p_c}\,, \end{aligned}$$

where f_c is the frequency of codon c in the current transcriptome of mRNAs present in the cell. First I note that τ_a is a fixed value which depends only on rates of translocation and accommodation of the amino acid into the nascent peptide chain. Thus, it is clearly independent of the tRNA concentration and tRNA bias in ternary complex. Similarly, τ_{off} is also independent of tRNA concentration and can be effectively considered as a constant. Hence, altering the value of T can only be done by either (a) affecting the time to bind any tRNA into the A-site of the ribosome (τ_{on}) which is inversely proportional to the *total* tRNA concentration, or (b) by altering the tRNA bias in the ternary complex which will change the values of p_c for all of the codons.

The goal is to minimize T by identifying the optimal tRNA bias, i.e. the optimal fractional numbers (e.g. $n_{pro1} = N_{pro1}/N$) of tRNA in ternary complex. With respect to the fractional number of 42 tRNAs n_i, the function that should be minimised is

$$g(n_i, \lambda) = \sum_c \frac{f_c}{p_c} + \lambda \left(\sum_i n_i - 1 \right)\,,$$

with λ the Lagrange multiplier. However, this function has no extrema due to the recognition of some codons by multiple tRNAs causing g to be over constrained. To illustrate this problem, consider the derivative of g with respect to the fractional

number of tRNA pro1, pro2, and pro3 in ternary complex (denote these as n_1, n_2, n_3) which each decode 1,2, and 3 codons respectively. The p_c terms for the four proline codons are

$$\begin{aligned} p_{cca} &= n_3 \\ p_{ccc} &= n_2 \\ p_{ccg} &= n_1 + n_3 \\ p_{ccu} &= n_2 + n_3 \,, \end{aligned} \qquad [1]$$

which gives the following partial derivatives of g

$$\begin{aligned} \frac{\partial g}{\partial n_1} &= -\frac{f_{ccg}}{p_{ccg}^2} + \lambda \\ \frac{\partial g}{\partial n_2} &= -\frac{f_{ccc}}{p_{ccc}^2} - \frac{f_{ccu}}{p_{ccu}^2} + \lambda \\ \frac{\partial g}{\partial n_3} &= -\frac{f_{cca}}{p_{cca}^2} - \frac{f_{ccg}}{p_{ccg}^2} - \frac{f_{ccu}}{p_{ccu}^2} + \lambda \,. \end{aligned} \qquad [2]$$

Setting these 3 derivatives to zero to solve for the minimum results in 3 coupled quadratic equations

$$\begin{aligned} \lambda &= \frac{f_{ccg}}{(n_1 + n_3)^2} \\ \lambda &= \frac{f_{ccu}}{(n_2 + n_3)^2} + \frac{f_{ccc}}{n_2^2} \\ 0 &= \frac{f_{ccu}}{(n_2 + n_3)^2} + \frac{f_{cca}}{n_3^2} \end{aligned}$$

Letting $x = n_3/n_2$, the last quadratic equation can be re-arranged to

$$1 + 2x + \left(1 + \frac{f_{ccu}}{f_{cca}}\right) x^2 = 0 \,.$$

Since $f_c \geq 0$ for all codons, then one can easily see that this quadratic equation has only imaginary solutions for $x = n_3/n_2$, implying that the three constraint conditions cannot be satisfied simultaneously. In general, this problem arises for any codon which is not decoded by a single tRNA, as is the case for the CCG and CCU codons in the proline example. If we instead had the situation where each sense codon is decoded by a unique tRNA, then $p_c = n_c$ for all codons and

$$\frac{\partial g}{\partial p_i} = -\frac{f_i}{n_i^2} + \lambda = 0 \,,$$

which must be true for all n_i when g is at an extrema. Solving for n_i

$$n_i^2 = \frac{f_i}{\lambda} \,,$$

and substituting into the constraint equation, one finds

$$\sum_c \sqrt{f_c} = \sqrt{\lambda} \,.$$

Since $n_i = p_i > 0$ for all codons, this implies that there is only one extrema for g which is given by

$$n_i = \frac{\sqrt{f_i}}{\sum_c \sqrt{f_c}} \,. \qquad [3]$$

A similar formula where the optimal total concentration of tRNAs decoding codon c was found to be proportional to the square root of f_c was also derived using a different theoretical approach by Ehrenburg and Kurland (22) as well as Berg and Kurland (23).

One solution to the constraint problem is to enforce that the ratio between tRNA isoacceptors in ternary complex decoding a particular codon is a constant. As an example, consider the three tRNA isoacceptors (pro1, pro2, and pro3) which decode the four proline codons. Again, let the fractional numbers of each be denoted by n_1, n_2, n_3. Then one can construct the ratios

$$r_i = \frac{n_i}{n_1 + n_2 + n_3} = \frac{N_i}{N_1 + N_2 + N_3} \,,$$

for each isoacceptor. Defining $n_{pro} = n_1 + n_2 + n_3$ allows one to write the fractional number of each tRNA isoacceptor as the ratio times the total fractional number of tRNAs that decode proline, $n_i = r_i n_{pro}$. Assuming that the ratios r_i are fixed constants and that only n_{pro} is variable, the probabilities in Eq. 1 become

$$\begin{aligned} p_{cca} &= r_3 n_{pro} \\ p_{ccc} &= r_2 n_{pro} \\ p_{ccg} &= (r_1 + r_3) n_{pro} \\ p_{ccu} &= (r_2 + r_3) n_{pro} \,. \end{aligned} \qquad [4]$$

This means that only one partial derivative with respect to n_{pro} needs to be taken, as opposed to three in Eq. 2, i.e.

$$\frac{\partial g}{\partial n_{pro}} = -\frac{f_{cca}}{p_{cca}^2} r_3 - \frac{f_{ccc}}{p_{ccc}^2} r_2 - \frac{f_{ccg}}{p_{ccg}^2}(r_1 + r_3) - \frac{f_{ccu}}{p_{ccu}^2}(r_2 + r_3) + \lambda \,.$$

Since p_{cca} etc. are all proportional to n_{pro}, one can directly solve the constraint equation for n_{pro} in terms of the constants r_i and λ and a exact solution for all of the tRNA fractions can be obtained.

Using this approach, I have calculated the tRNA fractions that would yield the optimal translation rate based on the codon usage (or codon bias) that has been measured in both Li *et al.* and Dong *et al.* (13, 24). Briefly, Li *et al.* used mRNA-seq to determine the average mRNA levels (in units of RPKM) that are present in exponentially growing *E. coli* cells. This information can be used to back construct a snapshot of the average transcriptome in the cell (see Methods in main text). Once a transcriptome is constructed, the codon usage can be compared with the codon usage which was computed separately by Dong *et al.* (24). Tab 3 in S1 Spreadsheet shows that the codon usage as measured by Dong *et al.* is in excellent agreement with the codon usage computed from the transcriptome that I have constructed using mRNA-seq data from Li *et al.* (13).

The codon bias was then used to determine the codon frequencies f_c and the tRNA fractions (cf. Table G) that would yield the optimal elongation rate. Tab 4 in S1 Spreadsheet gives the amino acid usage rates per second when the tRNA concentrations in ternary complex are fixed to those required for optimal translations. As an example, Table G gives the number of tRNAs in ternary complex for a growth rate of $\mu = 0.41$ h^{-1}. The numbers of tRNAs in ternary complex used by the model and adjusted to take into account tRNA abundances measured by Dong *et al* (24) are also given in Table G while Tab 5 in S1 Spreadsheet give the corresponding amino acid usage rates per second when tRNAs in ternary complex are fixed at these numbers.

5. Effect of tRNA Bias in Ternary Complex on tRNA Usage Rates

Given a codon (e.g. CCG) which is decoded by more then one tRNA isoacceptor (tRNA^{pro1} and tRNA^{pro3}), one might ask what would be the relative usage rates of each tRNA isoacceptor in decoding the CCG codon. More generally, since tRNA^{pro3} decodes three codons (CCA,CCG, and CCU), it would be useful to obtain the average usage rate of tRNA^{pro3} overall. To calculate this, denote the probability of decoding codon CCG by tRNA^{pro3} as P_{ccg}^3. Since CCG can be decoded by two tRNAs, this probability is simply

$$P_{ccg}^3 = \frac{N_3}{N_1 + N_3} \,,$$

where N_1 is the number of tRNA^{pro1} which are in ternary complex. The usage rate of tRNA^{pro3} in decoding the CCG codon is then

$$U_{ccg}^3 = \alpha P_{ccg}^3 f_{ccg} \,,$$

where f_{ccg} is the codon frequency of CCG in the transcriptome and α is a factor relating to the average translational speed of the ribosome so that U_{ccg}^3 has units of s^{-1}. Repeating the procedure for the other proline codons (CCA, CCC, and CCU), we obtain the total average usage rates of all three tRNAs as

$$\begin{aligned} U^1 &= \alpha \frac{N_1}{N_1 + N_3} f_{ccg} \\ U^2 &= \alpha \left[\frac{N_2}{N_2 + N_3} f_{ccu} + f_{ccc} \right] \\ U^3 &= \alpha \left[\frac{N_3}{N_1 + N_3} f_{ccg} + \frac{N_3}{N_2 + N_3} f_{ccu} + f_{cca} \right] \,. \end{aligned}$$

Since the CCA codon can only be decoded by tRNA^{pro3}, N_3 must be non-zero and we must enforce that $N_3 > 0$, with a similar argument for N_2. Let $x = N_1/N_3$ and $y = N_2/N_3 > 0$, then the usage rates can be rewritten as

$$\begin{aligned} U^1 &= \alpha \frac{x}{x+1} f_{ccg} \\ U^2 &= \alpha \left[\frac{y}{y+1} f_{ccu} + f_{ccc} \right] \\ U^3 &= \alpha \left[\frac{1}{x+1} f_{ccg} + \frac{1}{y+1} f_{ccu} + f_{cca} \right] \,. \end{aligned}$$

Finally, if we define β as

$$\beta = \alpha(f_{ccg} + f_{cca} + f_{ccc} + f_{ccu}) ,$$

with

$$r_{ccx} = \frac{f_{ccx}}{(f_{ccg} + f_{cca} + f_{ccc} + f_{ccu})} ,$$

then $U^1 + U^2 + U^3 = \beta$. Codon usage rates (per million) for the CCA, CCC, CCG, and CCU codons are 5085, 1011, 25949, and 5558, respectively (see Tab 3 in S1 Spreadsheet).

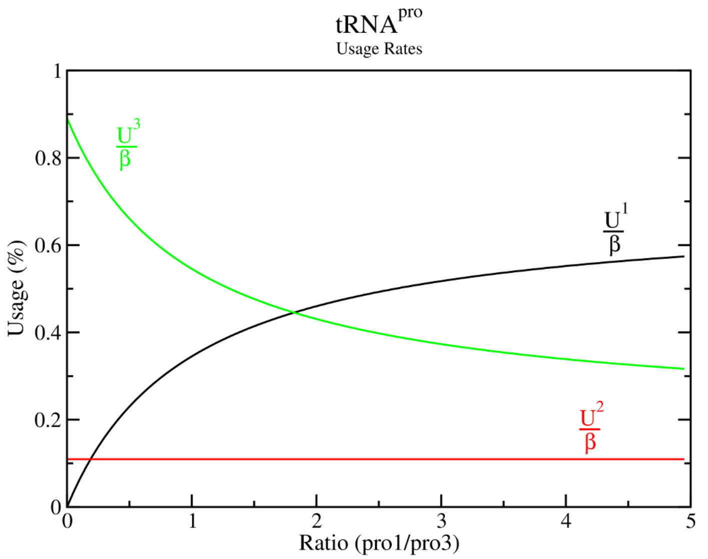

Fig H. Average tRNA usage rates for the three proline tRNA isoacceptors. Theoretical average tRNA usage rates for tRNA^{pro1} (black line), tRNA^{pro2} (red line) and tRNA^{pro3} (green line) are shown using a fixed ratio of tRNA^{pro2} to tRNA^{pro3} of y = 1.25 in ternary complex. The graph is plotted as a function of the ratio of tRNA^{pro1} to tRNA^{pro3} in ternary complex.

Fig H shows how the relative usage of tRNA^{pro1} vs tRNA^{pro3} changes as the amount of tRNA^{pro1} increases, assuming a fixed ratio of tRNA^{pro2} to tRNA^{pro3} of y = 1.25 in ternary complex. When the amount of tRNA^{pro1} in ternary complex is zero, then roughly 90% of proline codons are decoded by tRNA^{pro3} and 10% by tRNA^{pro2}. As the relative amount of tRNA^{pro1} increases relative to tRNA^{pro3}, the usage rate of tRNA^{pro1} steadily increases until it becomes the dominant tRNA that is used by the ribosome to decode proline codons. This effect is taken into account when fitting total tRNA numbers to the experimental measurements of Dong *et al.* (24) and for estimating the ratios for tRNA bias calculations in Eq. 4.

6. Estimation of Average E. Coli Cell Volumes at Different Growth Rates

Since I work with numbers of tRNAs and AARS enzymes, I need to know the volume of an average *E. coli* cell at various growth rates in order to fit to K_m values which are typically measured in units of μM. Several experimental groups have measured the dimensions of *E. coli* cells (25–28), which as noted by Bremer and Dennis, are typically done by either a particle counter (Coulter counter) or electron microscopy (16). Bremer and Dennis report that several experimental measurements done with the particle counter method determined average cell sizes of 0.83 and 1.12μm^3 for cells with doubling times of $\tau = 46$ and 28 min, respectively. They also reported electron microscopy experiments which gave average cell volumes of 0.63 and 1.20μm^3 for cells with doubling times of $\tau = 60$ and 24 min, respectively. The results of their analysis suggested that cell volume increases exponentially with growth rate, with values of 0.50, 0.63 and 1.20 μm^3 for cells with doubling times of 100, 60, and 24 min respectively.

In this work I have taken the results from Bremer and Dennis along with several experimental groups (25–28) and obtained a consensus average volume measurement for exponentially growing *E. coli* cells. The consensus values that I have used in this work are 0.45, 0.65, 0.90, and 1.95 μm^3 for cells with doubling times of 100, 60, 40, and 24 min respectively. These values are similar to those plotted in supplementary figure 7 of Belliveau *et al.* (29) which also obtains consensus average volumes for exponentially growing *E. coli* cells from an analysis of multiple experimental measurements.

7. Optimization Procedure for kcat, Km, and tRNA Numbers

When optimizing k_{cat} and K_m values of AARS enzymes for computer simulations we need to ideally choose optimal values that result in the simulations of tRNA charging and ribosome elongation also being consistent with a variety of additional

experimental observations. These include; (1) experimental measurements of the average total tRNA numbers in the cell, (2) proteomics data on the number of enzymes, (3) the percentages of charged tRNA in the cell, and (4) experimental measurements of average amino acid concentrations. Although there are 20 AARS enzymes, we are fortunate that each can be optimized separately as they are independent of each other. Although each AARS enzyme can be optimised independently, this optimization problem is still extraordinarily complex since we are attempting to match multiple experimental data points, each of which will effect the choice of k_{cat} and K_m values as well as have their own experimental errors. Moreover, as can be seen from the data on k_{cat} measurements from a variety of experimental groups (main text, Fig 6), there is a substantial range of potential values making it difficult to pinpoint a precise one to choose.

As a starting point, let us assume that ATP and amino acid are saturating on the AARS enzyme. Then we essentially wish to fit

$$r = \frac{k_{cat} T_u}{K_m^t + T_u} \, ,$$

where r is the turnover rate for the AARS enzyme and T_u is the quantity of *free* uncharged tRNA in the cell. Note that the turnover rate per enzyme r depends on the amino acid usage rate and the number of enzymes, which is experimentally measured *in vivo*. Similarly, k_{cat} and K_m are measured by *in vitro* kinetic assays. Finally, the free amount of uncharged tRNA in the cell will determine the charging ratio and be dependent on the total number of tRNAs in the cell. Thus, each variable in the equation essentially needs to be chosen in some way which minimizes the error to these experimental data points. To simplify things, I fix r for each enzyme using the consensus value from proteomics data (red lines in Fig F and Fig G), except for alaRS, proRS, and pheRS, which are lowered to get a better consensus fit with single-turnover transfer rates (k_{tran}) measured for the enzymes.

If one now supposes that we know the quantity of *free* uncharged tRNA in the cell T_u, then one can re-arrange the above equation to the straight line equation

$$k_{cat} = a K_m^t + r \, , \qquad [5]$$

where the slope of the line is given by $a = r/T_u$. Thus, given the amount of free uncharged tRNA in the cell and the turnover rate r, one can identify a pair of k_{cat} and K_m^t values on the line which minimize the error to experimental measurements. Hence, we can see that the critical (and most difficult) part of the fitting is the determination of the amount of free uncharged tRNA present in the cell.

Ideally, one would want to systematically vary the amount of free uncharged tRNA (T_u) as well as the amount of each tRNA in ternary complex (T_{tc}) for all 42 isoacceptors, looking for solutions which match the total amount of tRNA measured by Dong (24). The amount of uncharged free tRNA allows us to identify a best fit pair of k_{cat} and K_m^t which minimises their error with respect to experiment, while the amount of tRNA in ternary complex will set the amino acid usage rate and expected turnover rate. Then for each variation, the tRNA consumption rate can be calculated and an AARS enzyme model can be parameterised to reproduce k_{cat} and K_m. Then finally the parameterised model can be tested that it reproduces the expected kinetics. In practice however, the fitting procedure can only be partially automated since the computational complexity of systematically varying the free uncharged tRNA concentration and re-building and testing a parameterised AARS model each time is too high. I have set out the three stage procedure that I have used below.

Stage 1: Initial parameterisation of AARS enzymes

Step 1: Use the optimal tRNA numbers of each isoacceptor in ternary complex (Table G) to estimate the upper bound on; (1) the average rates of tRNA usage and, (2) the average number of tRNA bound to ribosomes (T_r). This is done by fixing ternary complex concentration in my ribosome translation model (19), then averaging the resulting tRNA usages over 30 minutes of steady-state translation. Tab 4 in S1 Spreadsheet gives the computed upper bound amino acid usage rates. For different growth rates, an assumption that 50% of the total tRNA is in ternary complex is used so that the elongation rate is nearly maximized following Fig E.

Step 2: Use the turnover rates calculated from step 1 to estimate the number of enzymes N using the consensus turnover rates from Fig F and Fig G (red lines). As a first approximation, assume that the amount of free charged tRNA (T_c) is zero and estimate the number of tRNA bound to AARS as $T_b = 0.75N$, which tends to be a very good starting estimate. The amount of free uncharged tRNA is then estimated using $T_u = T_t - T_{tc} - T_r - T_b$), where T_t is the total tRNA measured in Dong *et al.* (24), T_{tc} is the total tRNA in ternary complex, and T_r is the total bound to ribosome computed in step 1. Use the average experimental value for K_m^t and Eq. 5 to obtain a k_{cat} estimate. As an example calculation, at $\mu = 0.41$ h^{-1}, cysRS has (from Tab 8 in S1 Spreadsheet) $T_t = 1625$, $T_{tc} = 451$, and $T_r = 96$, with a consumption rate of 731.5 per sec (from Tab 4 in S2 spreadsheet). Using the consensus turnover rate of 1.6 for cysRS (Fig 5 in main text), the number of cysRS enzymes is $N = 457$ and $T_b = 343$. Thus, one expects that the number of free uncharged tRNA^{cys} is approximately $T_u = 735$, or 2.71 μM using a cell volume of 0.45 μm^3. Applying Eq. 5 with the average $K_m^t = 1.06$ for cysRS (see cysRS tab in S1 Spreadsheet) gives $k_{cat} = 2.2$ s^{-1}.

Step 3: Fit parameters for the AARS model using the procedure in section 1 of S1 Text assuming K_m^a and K_m^{atp} are the experimental average. Test that the model can support the turnover rate computed in Step 1 assuming free concentrations of AMP and pyrophosphate of $[AMP] = 250\mu$M and $[PP] = 500\mu$M (4, 30, 31). Note: k_{cat} may require a small increase at this stage to accommodate inhibition by AMP and pyrophosphate.

Stage 2: Refinement of tRNA numbers in ternary complex

Step 1: Adjust the number of tRNA in ternary complex by simulating the kinetics of all 20 enzymes undergoing constant tRNA consumption at the optimal rate. This returns target values for the amount of tRNA in ternary complex which will be the model fit (see Table G). Note this adjustment needs to be done since there are several highly expressed tRNAs (e.g. arg2 and leu1) that are have substantially more tRNA in ternary complex then is optimal.

Step 2: Repeat STAGE 1 procedure using the adjusted ternary complex numbers.

Stage 3: Refinement of AARS models and total tRNA numbers

Step 1: With models now constructed for each AARS based on adjusted ternary complex numbers, we can obtain a better estimate for the true amount of free uncharged tRNA. Using kinetic simulations of AARS kinetics with fixed tRNA consumption rates, fine adjustments are made to the total tRNA numbers in order to match Dong *et al.* and the overall % of tRNA that is charged.

Step 2: Construct a Michaelis–Menten model by using the k_{cat}, K_m values and enzyme numbers in Table I. The total tRNA numbers are adjusted computationally in the ribosome translation model (19) until the average amount of tRNA in ternary complex matches the target (see Tab 8 in S1 Spreadsheet for target tRNA numbers in ternary complex).

The results of the fitting procedure for k_{cat}, K_m, number of enzymes, and tRNA numbers are shown in Tables I and J. A direct comparison with measured tRNA numbers from Dong *et al.* (24) at a growth rate of $\mu = 0.41$ h^{-1} can be found in Table G, or in Tab 8 in S1 Spreadsheet for other growth rates. While most of the tRNAs deviate by less then 20% from the Dong measurements, there are a few outliers with $\approx$ 60% deviation. In particular tRNAs lys, asn, arg4, arg5, metM, pro3 and phe. Interestingly, as reported by Mohanty and Kushner (32), *E. coli* MG1655 expresses several tRNA as polycystronic transcripts which are then post-processed. For instance, ser3 (serV) and four copies of arg4 (argQ, argV, argY, argZ) are all expressed via a single polycistronic transcript. Since tRNA is a highly stable RNA in the cell (33), it is reasonable to expect that a ratio of arg2 / ser3 = 4 is maintained. Indeed, measurements by Dong *et al.* report values very close to this ratio. However, one copy of leu3 with two copies of metM, gln1, and gln2 are present on another polycistronic transcript suggesting a ratio of metM/leu3 $\approx$ 2. This ratio is not seen in the Dong measurements. However, I find that enforcement of this ratio gives much better results. This accounts for the high differences between the model fit and measurements seen for pro3, metM, his, gln1, and gln2 tRNAs. For tRNAs lys, asn, and phe, I observed that the total quantities of tRNA needed to increase in order to account for the large proportion of tRNAs that are in complex with ribosomes and AARS enzymes.

8. Theoretical tRNA Charging Fractions

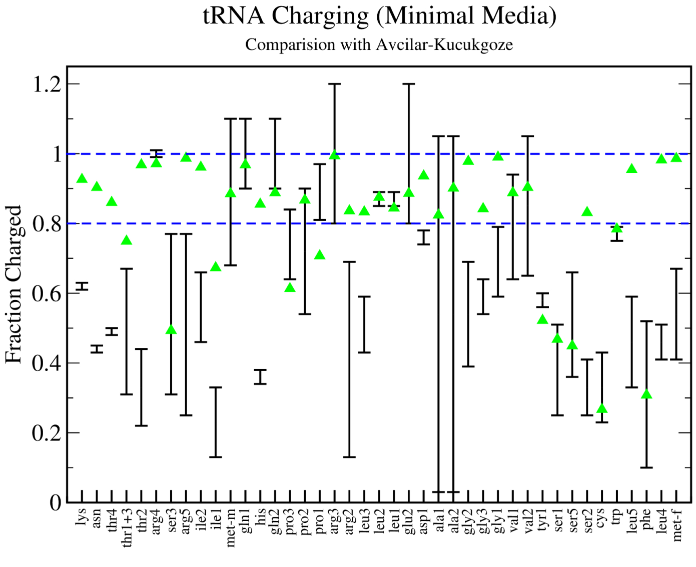

Fig I.**Theoretical and Experimental Estimates of tRNA Charging Fractions in Minimal Media.** The total fraction of tRNAs charged in exponentially growing *E. coli* cells. Black lines with error bars indicate the range of experimental measurement from Avcilar-Kucukgoze *et al.* (34), while green triangles give the results of the stochastic translational model with aminoacyl tRNA synthetase kinetics following the reaction scheme in Fig C and Fig D at a growth rate of $\mu = 0.41$ h^{-1}.

9. Tab Information in S1 Spreadsheet

A description of the data contained in each tab of the supplementary spreadsheet is as follows.

Tab A Classification of each AARS is given including; its class, the number of subunits, if burst kinetics has been observed, and if editing (pre or post transfer) has been identified.

Tab B Proteomics data for the twelve measurements used in this study. The PubMed ID (PMID) is given for each data source in columns R and S.

Tab C Experimental estimates of codon usage rates for exponentially growing *E. Coli* with doubling times of 60 and 21 min. Columns H-K report number per second. Column A-D are explicit data from Dong. Data is obtained from Dong *et al.* and ribosome profiling from Li *et al.*

Tab D Estimated upper bound on the AARS turnover rates at four different *E. coli* growth rates. The upper bound is determined by enforcing an elongation rate of 19.1 aa/sec in the translation model.

Tab E Simulated AARS turnover rates at four different *E. coli* growth rates. The rate is determined by simulating the maximum elongation rate possible in the translation model which will be dependant on the volume of the cell, and fraction of tRNA in ternary complex.

Tab F Fitting of K_m and k_{cat} values for each AARS to the AARS turnover rates computed in tab T5. E(exp) reports the experimental consensus on the number of AARS enzymes, E(fit) corresponds to the fitted number used in the model. [Tu] Predicted and [Tu] Simulated report the predicted amount of free un-charged tRNA (based on the MM equation) and the simulated free un-charged tRNA that is computed using the full kinetic model for the AARS.

Tab G Experimental measurements of the number of tRNAs in exponentially growing *E. coli* cells from Dong *et al.*

Tab H Fitting of tRNA numbers to the experimental measurements of Dong *et al.* Column Dong is the number reported by Dong et al., while Tot (fit) is the number used in the model. The Ribo column reports the number of tRNAs bound to all translating ribosomes in the cell and is estimated from simulations. Columns AE-AL give the fitted numbers color coded by their error to the dong measurements.

Tab I Amino acid concentrations in the cell from three different measurements along with experimental measurements of the tRNA charging fractions. PubMed ID of the data sources are given in the color coded boxes.

Tab J Experimental *in vitro* measurements of cysRS tRNA charging kinetics. PMID column lists the PubMed ID of the reference data source, along with k_{cat} and K_m for different substrates. Experimental measurements of substrate binding constants are also given along with the reference source of the data as PMID. Columns L-P give the average of all experimental data and the model fit. Substrate binding constants used in the model are also given.

Table A. Classification and Properties of Aminoacyl tRNA Synthetases from *E. coli*. For each of the 23 genes encoding an aminoacyl tRNA synthetase, the class, number of subunits which make up the functional enzyme, and if the enzyme has been observed to have burst kinetics or editing, is shown.

AARS	Gene	Class	Subunits	Burst	tRNA Dep.	Editing
cysRS	cysS	I	1	Y		
argRS	argS	I	1	Y	Y	
valRS	valS	I	1	Y		Y
ileRS	ileS	I	1			Y
leuRS	leuS	I	1	Y		Y
gluRS	gltX	I	1	Y	Y	
glnRS	glnS	I	1	Y	Y	
metRS	metG	I	2	Y		Y
tyrRS	tyrS	I	2	Y		
trpRS	trpS	I	2	Y		
serRS	serS	II	2			Y
thrRS	thrS	II	2			Y
proRS	proS	II	2			Y
hisRS	hisS	II	2			
aspRS	aspS	II	2			
asnRS	asnS	II	2			
lysRS	lysS	II	2			Y
alaRS	alaS	II	2			Y
lysRS*	lysU	II	2			Y
glyRSα	glyS	II	2			
glyRSβ	glyQ	II	2			
pheRSα	pheS	II	2			Y
pheRSβ	pheT	II	2			Y

Table B. Kinetic parameters for Class I aminoacyl tRNA synthetase models. Labels of the reactions correspond to those shown in Fig C.

Enzyme	k_{1f} $1/\mu$Ms	k_{1b} $1/s$	k_{2f} $1/\mu$Ms	k_{2b} $1/s$	k_{3f} $1/\mu$Ms	k_{3b} $1/s$	k_{4f} $1/\mu$Ms	k_{4b} $1/s$	k_{5f} $1/s$	k_{5b} $1/s$	k_{6f} $1/\mu$Ms	k_{6b} $1/s$	k^a_{0f} $1/\mu$Ms	k^a_{0b} $1/s$	k^b_{0f} $1/\mu$Ms	k^b_{0b} $1/s$
cysRS	0.24	14.4	6.00	3480	6.00	2320	0.24	9.6	160	400	6.00	3000	46.0	138	46.0	138
argRS	2.40	180	6.00	720	6.00	720	2.40	180	0	0	0	0	40.0	184	40.0	36.8
valRS	1.00	138	6.00	3000	6.00	2000	1.00	92	140	400	6.00	3000	80.0	30	80.0	30
ileRS	2.20	15	6.00	2400	6.00	2400	2.20	15	150	400	6.00	3000	120	138	120	138
leuRS	1.20	90	6.00	3300	6.00	3300	1.20	90	135	350	6.00	3000	40.0	168	40.0	56
gluRS	0.15	27	3.40	3740	3.40	3740	0.15	27	0	0	0	0	80.0	64	80.0	64
glnRS	0.20	216	5.00	3400	5.00	3400	0.20	216	0	0	0	0	120	42	120	42
tyrRS	0.60	15	4.20	3360	4.20	2688	0.60	12	160	320	6.00	3000	42.0	48	42.0	48
trpRS	0.30	13.5	8.00	2500	8.00	2500	0.30	13.5	320	400	6.00	3000	45.0	117	45.0	117

Enzyme	k^t_{1f} $1/\mu$Ms	k^t_{1b} $1/s$	k^t_{2f} $1/\mu$Ms	k^t_{2b} $1/s$	k^t_{3f} $1/\mu$Ms	k^t_{3b} $1/s$	k^t_{4f} $1/\mu$Ms	k^t_{4b} $1/s$	k^t_{5f} $1/s$	k^t_{5b} $1/s$	k^t_{6f} $1/\mu$Ms	k^t_{6b} $1/s$	k^c_{0f} $1/\mu$Ms	k^c_{0b} $1/s$	k^d_{0f} $1/\mu$Ms	k^d_{0b} $1/s$
cysRS	0.24	14.4	6.00	3480	6.00	3480	0.24	14.4	160	400	0.01	5.0	46.0	138	30.0	135
argRS	2.40	36	6.00	3600	6.00	3600	2.40	36	270	400	0.01	5.0	15.0	345	40.0	184
valRS	1.00	138	6.00	3000	6.00	3000	1.00	138	140	400	0.01	5.0	80.0	30	80.0	45
ileRS	2.20	15	6.00	2400	6.00	2400	2.20	15	150	400	0.01	5.0	120	138	120	138
leuRS	1.20	30	2.20	3630	2.20	3630	1.20	30	135	350	0.01	5.0	15.0	189	40.0	168
gluRS	0.15	27	3.40	3740	3.40	3740	0.15	27	140	400	0.01	5.0	80.0	64	80.0	64
glnRS	0.20	216	5.00	3400	5.00	3400	0.20	216	140	400	0.01	5.0	120	42	120	42
tyrRS	0.60	15	4.20	3630	4.20	2688	0.60	12	160	320	0.01	5.0	42.0	48	42.0	48
trpRS	0.30	13.5	8.00	2500	8.00	2500	0.30	13.5	320	400	0.01	5.0	45.0	117	45.0	117

Enzyme	k_{7f} $1/s$	k_{7b} $1/s$	k_{8f} $1/s$	k_{8b} $1/s$	k_{9f} $1/\mu$Ms	k_{9b} $1/s$	k_{10f} $1/\mu$Ms	k_{10b} $1/s$	k_{11f} $1/\mu$Ms	k_{11b} $1/s$	k^e_{0f} $1/\mu$Ms	k^e_{0b} $1/s$
cysRS	114	400	17.3	60.7	6.00	3000	1.00	2400	12.3	3.70	30.0	135
argRS	340	400	74.8	88	6.00	3000	1.00	2400	40.0	29.0	40.0	184
valRS	240	400	30.7	51.2	6.00	3000	1.00	2400	20.0	16.0	80.0	45
ileRS	38	114	7.8	23.4	6.00	3000	1.00	2400	100	65.0	120	138
leuRS	289	340	62.9	74	6.00	3000	1.00	2400	10.0	15.7	40.0	168
gluRS	220	400	28.0	51	6.00	3000	1.00	2400	30.0	8.1	80.0	64
glnRS	220	400	28.0	51	6.00	3000	1.00	2400	12.0	7.4	120	42
tyrRS	140	280	32.0	64	6.00	3000	1.00	2400	16.0	8.7	42.0	48
trpRS	175	350	44.0	88	6.00	3000	1.00	2400	10.0	3.3	45.0	117

Table C. Kinetic parameters for Class II aminoacyl tRNA synthetase models. Part 1 of 2. Labels of the reactions correspond to those shown in Fig D.

Enzyme	k_{1f} 1/μMs	k_{1b} 1/s	k_{2f} 1/μMs	k_{2b} 1/s	k_{3f} 1/μMs	k_{3b} 1/s	k_{4f} 1/μMs	k_{4b} 1/s	k_{5f} 1/s	k_{5b} 1/s	k_{6f} 1/μMs	k_{6b} 1/s	k^a_{0f} 1/μMs	k^a_{0b} 1/s	k^b_{0f} 1/μMs	k^b_{0b} 1/s
metRS	1.40	70.0	4.00	3600	4.00	2880	1.40	56.0	170	320	6.00	3000	120	84	120	84
serRS	0.30	18.0	6.00	3600	6.00	2880	0.30	14.4	160	400	6.00	3000	50.0	87.5	50.0	87.5
thrRS	0.24	60.0	6.00	3600	6.00	2250	0.24	37.5	180	400	6.00	3000	50.0	130	50.0	130
proRS	0.25	100	8.00	1600	8.00	1600	0.25	100	160	400	6.00	3000	42.0	84	42.0	84
hisRS	2.00	150	3.40	3600	3.40	2880	2.00	120	300	500	6.00	3000	90.0	150	90.0	150
aspRS	0.20	174	4.60	3450	4.60	3450	0.20	174	60	150	6.00	3000	80.0	140	80.0	140
asnRS	0.24	7.2	4.60	3496	4.60	3496	0.24	7.2	160	360	6.00	3000	40.0	48	40.0	48
lysRS	2.00	180	6.00	3600	6.00	2400	2.00	120	100	250	6.00	3000	58.0	125	58.0	125
alaRS	0.70	280	8.00	1600	8.00	1600	0.70	280	180	400	6.00	3000	92.0	184	92.0	184
glyRS	0.32	56.0	6.00	3600	6.00	3600	0.32	56.0	160	400	6.00	3000	80.0	100	80.0	100
pheRS	2.00	270	2.80	3600	2.80	2400	2.00	180	300	500	6.00	3000	37.0	44	37.0	44

Enzyme	k^t_{1f} 1/μMs	k^t_{1b} 1/s	k^t_{2f} 1/μMs	k^t_{2b} 1/s	k^t_{3f} 1/μMs	k^t_{3b} 1/s	k^t_{4f} 1/μMs	k^t_{4b} 1/s	k^t_{5f} 1/s	k^t_{5b} 1/s	k^t_{6f} 1/μMs	k^t_{6b} 1/s	k^c_{0f} 1/μMs	k^c_{0b} 1/s	k^d_{0f} 1/μMs	k^d_{0b} 1/s
metRS	1.40	70.0	4.00	3600	4.00	2880	1.40	56.0	170	320	6.00	3000	120	84	120	84
serRS	0.30	18.0	6.00	3600	6.00	2880	0.30	14.4	25.0	12.5	0.01	5.0	50.0	87.5	50.0	87.5
thrRS	0.24	60.0	6.00	3600	6.00	2250	0.24	37.5	29.0	19.0	6.00	3000	50.0	130	50.0	130
proRS	0.25	100	8.00	1600	8.00	1600	0.25	100	16.0	12.0	6.00	3000	42.0	84	42.0	84
hisRS	2.00	150	3.40	3600	3.40	2880	2.00	120	19.0	9.60	6.00	3000	90.0	150	90.0	150
aspRS	0.20	174	4.60	3450	4.60	3450	0.20	174	40.0	20.0	6.00	3000	80.0	140	80.0	140
asnRS	0.24	7.2	4.60	3496	4.60	3496	0.24	7.2	16.0	12.0	6.00	3000	40.0	48	40.0	48
lysRS	2.00	180	6.00	3600	6.00	2400	2.00	120	32.0	22.4	6.00	3000	58.0	125	58.0	125
alaRS	0.70	280	8.00	1600	8.00	1600	0.70	280	22.5	22.5	6.00	3000	92.0	184	92.0	184
glyRS	0.32	56.0	6.00	3600	6.00	3600	0.32	56.0	70.0	35.0	6.00	3000	80.0	100	80.0	100
pheRS	2.00	270	2.80	3600	2.80	2400	2.00	180	18.0	3.0	6.00	3000	37.0	44	37.0	44

Table D. Kinetic parameters for Class II aminoacyl tRNA synthetase models. Part 2 of 2. Labels of the reactions correspond to those shown in Fig D.

Enzyme	k_{7f} 1/s	k_{7b} 1/s	k_{8f} 1/s	k_{8b} 1/s	k_{9f} 1/μMs	k_{9b} 1/s	k_{10f} 1/μMs	k_{10b} 1/s	k_{11f} 1/μMs	k_{11b} 1/s	k_{12f} 1/μMs	k_{12b} 1/s	k^e_{0f} 1/μMs	k^e_{0b} 1/s	k^f_{0f} 1/μMs	k^f_{0b} 1/s
metRS	135	243	42.0	75.6	6.00	3000	1.00	2400	2.30	50.6	6.00	1800	120	84	120	84
serRS	180	360	26.0	52.0	6.00	3000	1.00	2400	0.30	18.0	6.00	360	60.0	21	50.0	87.5
thrRS	180	306	17.6	30.0	6.00	3000	1.00	2400	2.40	144	6.00	2100	50.0	38	50.0	38
proRS	80	240	15.0	45.0	6.00	3000	1.00	2400	0.25	70.0	8.00	1600	42.0	25.2	42.0	25.2
hisRS	115	383	20.0	67.0	6.00	3000	1.00	2400	4.00	70.0	6.00	2400	90.0	45	90.0	45
aspRS	180	360	25.0	50.0	6.00	3000	1.00	2400	0.80	51.2	4.60	1470	80.0	28	80.0	28
asnRS	120	360	15.5	46.5	6.00	3000	1.00	2400	0.24	7.2	6.00	1500	40.0	16	40.0	16
lysRS	160	320	30.0	60.0	6.00	3000	1.00	2400	4.00	64.0	6.00	240	58.0	35	58.0	35
alaRS	140	350	22.3	55.8	6.00	3000	1.00	2400	0.70	84.0	8.00	1600	92.0	83	92.0	83
glyRS	180	360	60.0	120	6.00	3000	1.00	2400	0.32	56.0	6.00	600	80.0	20	80.0	20
pheRS	120	360	18.0	54.0	6.00	3000	1.00	2400	3.00	39.0	6.00	1800	37.0	4.4	37.0	4.4

Enzyme	k_{13f} 1/μMs	k_{13b} 1/s	k_{14f} 1/μMs	k_{14b} 1/s	k_{15f} 1/s	k_{15b} 1/s	k_{16f} 1/μMs	k_{16b} 1/s	k_{17f} 1/μMs	k_{17b} 1/s	k_{18f} 1/μMs	k_{18b} 1/s
metRS	6.00	1800	2.30	50.6	67.0	10.0	6.00	3000	12.0	20	12.0	20
serRS	6.00	360	0.30	18.0	25.0	12.5	6.00	3000	50.0	50	50.0	50
thrRS	6.00	2100	2.40	144	39.0	19.0	6.00	3000	50.0	70	50.0	70
proRS	8.00	1600	0.25	70.0	16.0	12.0	6.00	3000	42.0	84	42.0	84
hisRS	6.00	2400	4.00	70.0	30.0	15.0	6.00	3000	50.0	60	50.0	60
aspRS	4.60	1470	0.80	51.2	40.0	20.0	6.00	3000	80.0	140	80.0	140
asnRS	6.00	1500	0.24	7.2	16.0	12.0	6.00	3000	40.0	40	40.0	40
lysRS	6.00	240	4.00	64.0	64.0	16.0	6.00	3000	58.0	125	58.0	125
alaRS	8.00	1600	0.70	84.0	60.0	22.5	6.00	3000	92.0	230	92.0	230
glyRS	6.00	600	0.32	56.0	70.0	35.0	6.00	3000	120	240	120	240
pheRS	6.00	1800	3.00	39.0	18.0	3.0	6.00	3000	40.0	80	40.0	80

Table E. Estimates of AARS Activity *in vivo*. For each of the AARS enzymes, the activity (i.e. tRNA turnover rate r) of a single enzyme is estimated in *E. coli* cells growing at $\mu = 0.69$ h^{-1} based on amino acid usage and the number of AARS. Amino acid usage for the Mori *et al.* data (11) was calculated computationally using a model of *in vivo* translation (19) and the fractional amount of tRNAs in ternary complex needed for optimal translation, while the amino acid usage for Jakubowski was taken from their experimental amino acid radio-labelling measurements (9).

		Mori et al.(11)			Jakubowski et al. (9)		
Protein	Gene	N. Pro.	AA Use (s^{-1})	r (s^{-1})	N. Enz.	AA Use (s^{-1})	r (s^{-1})
cysRS	cysS	826	1606	1.94		4600	
argRS	argS	916	14491	17.75	691	6770	9.79
valRS	valS	1553	19407	12.49	536	14050	26.21
ileRS	ileS	2061	13844	6.71	1226	15270	13.45
leuRS	leuS	1903	18583	9.76	875	20250	23.14
gluRS	gltX	2194	15534	7.08	873	1730	1.98
glnRS	glnS	1518	8337	5.49	1013	4980	4.91
metRS	metG	1732	6342	7.32		7630	
tyrRS	tyrS	1686	5486	6.50		1920	
trpRS	trpS	2475	1704	1.37		3150	
serRS	serS	1725	11066	12.83		51090	
thrRS	thrS	2807	12120	8.63	527	25210	47.84
proRS	proS	1969	8501	8.63		10030	
hisRS	hisS	934	4324	9.25		4030	
aspRS	aspS	1636	12104	14.79		15930	
asnRS	asnS	3604	8424	4.67		2100	
lysRS	lysS	2435	16038	13.17	425	11850	27.88
alaRS	alaS	1627	22820	28.05		13390	
lysRS*	lysU	278					
glyRSα	glyS	1744	18848	21.61	682	10210	14.97
glyRSβ	glyQ	1614	18848	23.35			
pheRSα	pheS	1319	7519	11.40	992	9950	10.03
pheRSβ	pheT	1819	7519	8.26			

Table F. Average Number of Proteins, tRNA, and Ribosomes in *E. coli* Cells at Different Growth Rates. Protein numbers are taken from proteome measurements from several groups (11–13) and reported here as number per cell. The specific data for each growth rate are; $\mu = 0.41$ h^{-1} Valgepea *et al.* 2013 (12), $\mu = 0.69$ h^{-1} and $\mu = 1.04$ h^{-1} Mori *et al.* 2021 (11), $\mu = 1.98$ h^{-1} Li *et al.* 2014 (13). The number of tRNA and Ribosomes (per cell) are taken from Dennis and Bremer (16). The doubling time in minutes is computed from the growth rate using $\tau = 60\ln(2)/\mu$.

Protein	Gene	$\mu = 0.41$ h^{-1} $\tau = 100$	$\mu = 0.69$ h^{-1} $\tau = 60$	$\mu = 1.04$ h^{-1} $\tau = 40$	$\mu = 1.98$ h^{-1} $\tau = 21$
Ribo		8000	15000	26000	73000
tRNA		74000	139000	241000	680000
cysRS	cysS	609	826	1409	4958
argRS	argS	733	916	1457	4901
valRS	valS	1179	1553	2502	8921
ileRS	ileS	2135	2061	2832	6143
leuRS	leuS	2706	1903	2534	8069
gluRS	gltX	2751	2194	2980	13429
glnRS	glnS	2076	1518	2113	8204
metRS	metG	1618	1732	2415	6555
tyrRS	tyrS	1447	1686	3661	7986
trpRS	trpS	1975	2475	3764	7805
serRS	serS	2948	1725	3241	11805
thrRS	thrS	1222	2807	4091	12096
proRS	proS	4060	1969	2415	12156
hisRS	hisS	1438	934	1956	3774
aspRS	aspS	2513	1636	2993	8307
asnRS	asnS	4035	3604	5615	17436
lysRS	lysS	4498	2435	3204	15890
alaRS	alaS	2000	1627	2962	8493
lysRS*	lysU	117	278	372	1644
glyRSα	glyS	2871	1744	2499	12974
glyRSβ	glyQ	2313	1614	2067	12365
pheRSα	pheS		1319	2443	8935
pheRSβ	pheT	1579	1819	3324	8573
EfTu	tufA	39582	51042	68208	289537
EfTu	tufB	39581	51105	68292	289537
EfTs	tfs	16181	15894	26017	95122
EfG	fusA	18213	15065	25810	84151
Inf1	infA	4843	5694	9220	30461
Inf2	infB	2856	2493	4082	13806
Inf3	infC	4876	4765	7890	22624
RRF	frr	5420	5237	7112	23316
RF1	prfA		184	341	1059
RF2	prfB		108	110	10031
RF3	prfC	614	874	1088	7808

Table G. Average number of tRNAs per cell and their codon recognition. Data for the number of tRNAs that are in free ternary complex at a growth rate of $\mu = 0.41$ h^{-1} ($\tau = 100$ min). The numbers that give the optimal translation rate have been computed from Eq. 3 and the procedure outlined in section 4, and the total tRNA numbers are taken from Dong *et al.* (24). The values used in the model are the result of the fitting procedure in section 7.

		Ternary Complex			Total tRNA		
tRNA	Codon Rec.	Opt. Trans.	Model	Error	Dong	Model	Error
Lys	AAA,AAG	1424	1429	0.00	2264	3210	0.41
Asn	AAC,AAU	1028	914	0.12	1285	2370	0.84
Thr4	ACA,ACU,ACG	629	675	0.07	1055	1216	0.15
Thr1+3	ACC,ACU	728	541	0.34	1378	1552	0.12
Thr2	ACG	353	449	0.27	666	541	0.18
Arg4	AGA	143	246	0.72	782	293	0.62
Ser3	AGC,AGU	641	641	0.00	1462	1224	0.16
Arg5	AGG	72	147	1.04	522	165	0.68
Ile2	AUA	139	271	0.94	224	320	0.42
Ile1	AUC,AUU	1319	1337	0.01	3722	3919	0.05
MetM	AUG	813	795	0.02	862	1540	0.78
Gln1	CAA	471	1156	1.45	905	1540	0.70
His	CAC,CAU	739	536	0.37	729	1166	0.60
Gln2	CAG	904	660	0.37	1025	1720	0.67
Pro3	CCA,CCU,CCG	393	420	0.07	629	1166	0.85
Pro2	CCC,CCU	495	509	0.03	836	708	0.15
Pro1	CCG	524	560	0.07	812	1165	0.43
Arg3	CGG	138	1067	6.73	869	1166	0.34
Arg2	CGU,CGC,CGA	1338	2150	0.61	4842	4896	0.01
Leu3	CUA,CUG	179	320	0.79	829	680	0.17
Leu2	CUC,CUU	545	550	0.01	1155	980	0.15
Leu1	CUG	1206	2080	0.72	4966	4322	0.13
Glu2	GAA,GAG	1403	1488	0.06	5189	4206	0.18
Asp1	GAC,GAU	1236	1223	0.01	2707	2586	0.04
Ala1B	GCU,GCA,GCG	1586	1607	0.01	3906	3919	0.01
Ala2	GCC	597	490	0.22	706	810	0.15
Gly2	GGA,GGG	279	1008	2.61	1055	1216	0.15
Gly3	GGC,GGU	1493	1540	0.03	5066	3981	0.21
Gly1a	GGG	163	705	3.32	1336	800	0.40
Val1	GUA,GUG,GUU	1364	1510	0.11	4020	3404	0.15
Val2ab	GUC,GUU	524	574	0.09	1462	1165	0.20
Tyr1+2	UAC,UAU	834	834	0.00	2087	2110	0.01
Ser1	UCA,UCU,UCG	818	763	0.07	1851	1420	0.23
Ser5	UCC,UCU	416	394	0.06	865	1112	0.28
Ser2	UCG	160	314	0.96	346	367	0.06
Cys	UGC,UGU	451	498	0.10	1625	1315	0.19
Trp	UGG	463	496	0.07	925	943	0.02
Leu5	UUA,UUG	336	1033	2.07	1155	1315	0.14
Phe	UUC,UUU	976	976	0.00	1199	2134	0.78
Leu4	UUG	650	1512	1.32	2108	1744	0.17

Table H. Concentrations of Amino Acids in *E. coli*. Experimental measurements of intracellular amino acid concentrations are shown for Bennett *et al.* (4), and minimal media (MM) and minimal media plus amino acids (MM+AA) from Avcilar-Kucukgoze (34). The values used in the model for the MM and MM+AA scenarios are shown in the final two columns. *(Combined concentration of ile+leu was measured to be 300 μM). (nd = not determined)

	Bennett	Avcilar-Kucukgoze		Model	
AA		MM	MM+AA	MM	MM+AA
cys	nd	nd	nd	30	300
arg	570	nd	nd	570	570
val	4200	185	3000	4200	4200
ile	80*	9	290	80	300
leu	220*	nd	nd	220	360
glu	96000	nd	nd	96000	96000
gln	3800	nd	210	3800	3800
met	150	nd	nd	150	160
tyr	29	11	210	29	270
trp	12	65	770	20	220
ser	68	57	4000	68	4000
thr	180	8	340	180	450
pro	390	nd	nd	390	2850
his	68	8	130	68	160
asp	4200	nd	nd	4200	4200
asn	510	2	230	510	510
lys	410	24	250	410	410
ala	2600	27	1210	2600	2600
gly	800	62	360	800	800
phe	18	12	270	20	270

Table I. Optimized numbers of Aminoacyl tRNA Synthetases in Exponentially Growing *E. coli* and optimized k_{cat} and K_m values. The optimized average number of AARS enzymes are shown for different growth rates, $\mu = 0.41$ h^{-1} ($\tau = 100$ min), $\mu = 0.69$ h^{-1} ($\tau = 60$ min), $\mu = 1.04$ h^{-1} ($\tau = 40$ min), and $\mu = 1.73$ h^{-1} ($\tau = 24$ min). Corresponding optimized k_{cat} and Michaelis–Menten parameters for tRNA (K_m^t) and amino acid (K_m^a) are given in the right hand columns.

	Optimized Numbers				Optimized Kcat/Km		
AARS	$\mu = 0.41$	$\mu = 0.69$	$\mu = 1.04$	$\mu = 1.73$	k_{cat} (s^{-1})	K_m^t (μM)	K_m^a (μM)
cysRS	440	895	1610	3800	2.9	1.05	16.1
argRS	450	857	1540	3400	20.2	1.76	15.1
valRS	1050	2100	3675	8140	10.6	0.41	42.0
ileRS	1140	2240	3945	9000	7.2	1.18	5.8
leuRS	1050	2115	3870	8900	11.2	1.17	15.6
gluRS	1350	2750	4860	10800	6.2	0.27	72.3
glnRS	850	1800	3265	7520	5.8	0.16	188
metRS	480	1020	1860	4180	10.7	0.75	8.1
tyrRS	500	1030	1840	4265	6.6	0.46	14.2
trpRS	550	1210	2260	5380	3.1	0.29	11.4
serRS	780	1640	3030	6800	8.7	0.39	56.0
thrRS	800	1600	2890	6510	9.6	0.72	24.1
proRS	790	1530	2750	6400	6.7	0.48	149
hisRS	400	800	1450	3330	9.2	0.41	7.9
aspRS	600	1200	2050	4600	12.5	0.47	38.8
asnRS	960	2015	3660	8260	5.8	0.52	34.6
lysRS	890	1450	2575	5690	16.3	0.94	8.8
alaRS	1000	1800	3260	7300	13.2	0.91	47.1
glyRS	480	930	1660	3650	24.7	0.68	140
pheRS	610	1250	2280	5185	7.7	0.35	9.1

Table J. Optimized numbers of tRNAs per cell and their codon recognition. The optimized total tRNA numbers are shown for the Michaelis–Menten model and the full kinetic model where AARS enzymes are modelled as having reaction scheme according to Fig C for Class I enzymes or Fig D for class II enzymes.

		Michaelis–Menten				Full Model			
tRNA	Codon Rec.	$\mu = 0.41$	$\mu = 0.69$	$\mu = 1.04$	$\mu = 1.73$	$\mu = 0.41$	$\mu = 0.69$	$\mu = 1.04$	$\mu = 1.73$
Lys	AAA,AAG	2759	5575	10627	23872	3210	6322	10980	23119
Asn	AAC,AAU	1805	3384	6509	14766	2370	4675	8324	18084
Thr4	ACA,ACU,ACG	1113	2200	4046	9199	1216	2399	4187	9108
Thr1+3	ACC,ACU	1348	2538	4532	10212	1552	2988	4979	10548
Thr2	ACG	533	1077	2076	4757	541	1107	2016	4539
Arg4	AGA	288	564	1190	2730	293	578	1153	2604
Ser3	AGC,AGU	961	1846	3309	7541	1224	2414	4213	9327
Arg5	AGG	165	323	689	1580	165	325	660	1496
Ile2	AUA	301	620	1142	2708	320	661	1154	2679
Ile1	AUC,AUU	2297	4565	9050	21003	3919	7731	13310	28748
MetF	AUG	2364	5132	7809	21870	2364	5132	7809	21870
MetM	AUG	1366	2618	4953	11230	1540	3078	5372	11729
Gln1	CAA	1377	2764	5089	11410	1540	3078	5372	11729
His	CAC,CAU	936	1829	3556	8237	1166	2300	4014	8881
Gln2	CAG	1155	2254	4448	10142	1720	3433	5992	13100
Pro3	CCA,CCU,CCG	862	1753	3317	7643	1166	2300	4014	8881
Pro2	CCC,CCU	648	1342	2644	6038	708	1450	2702	6042
Pro1	CCG	935	1880	3608	8337	1165	2316	4131	9173
Arg3	CGG	1186	2280	4167	9398	1166	2300	4014	8881
Arg2	CGU,CGC,CGA	4475	8880	16261	37500	4896	9656	16852	37308
Leu3	CUA,CUG	544	1078	2083	4665	680	1361	2375	5179
Leu2	CUC,CUU	824	1671	3277	7402	980	2012	3544	7838
Leu1	CUG	3441	6740	12992	28963	4322	8506	14780	31841
Glu2	GAA,GAG	2829	5483	10707	24536	4206	8291	14471	32464
Asp1	GAC,GAU	2294	4500	8982	20966	2586	5104	8908	19696
Ala1B	GCU,GCA,GCG	3689	8508	15144	35209	3919	7731	13310	28748
Ala2	GCC	787	1775	3429	8008	810	1689	3016	6747
Gly2	GGA,GGG	1181	2354	4312	9632	1216	2399	4187	9108
Gly3	GGC,GGU	3456	7184	13180	30997	3981	7853	13706	30817
Gly1a	GGG	790	1565	2841	6329	800	1578	2754	5983
Val1	GUA,GUG,GUU	2780	5387	10578	24202	3404	6700	11694	25926
Val2ab	GUC,GUU	969	1931	3767	8627	1165	2324	4056	8873
Tyr1+2	UAC,UAU	1586	3060	5942	13225	2110	4154	7250	15620
Ser1	UCA,UCU,UCG	1109	2187	3915	8900	1420	2808	4850	10711
Ser5	UCC,UCU	747	1417	2483	5637	1112	2113	3641	7884
Ser2	UCG	349	732	1297	2970	367	760	1307	2952
Cys	UGC,UGU	991	1903	3499	7801	1315	2594	4528	9996
Trp	UGG	689	1366	2694	6200	943	1920	3536	7977
Leu5	UUA,UUG	1231	2386	4470	10048	1315	2594	4528	9996
Phe	UUC,UUU	1693	3352	6450	14677	2134	4210	7320	15926
Leu4	UUG	1695	3335	6113	13796	1744	3440	6004	13283

Table K. Model predictions of AARS Activity *in vivo*. For each of the AARS enzymes, the activity (i.e. tRNA turnover rate) of a single enzyme r per second is estimated in *E. coli* cells growing at $\mu = 1.04$ h^{-1} ($\tau = 40$ min) based on amino acid usage and the number of AARS. Amino acid usage for the data in this work was calculated computationally using a model of *in vivo* translation (19), while the amino acid usage and AARS numbers for Choi and Covert were taken from supplementary material Tables 8 and S3 (10).

	This work			Choi and Covert (10)		
AARS	N. Enz.	AA Use (s^{-1})	r (s^{-1})	N. Enz.	AA Use (s^{-1})	r (s^{-1})
cysRS	1610	2714.8	1.68	606	2696.6	4.44
argRS	1540	24209.7	15.72	625	20752.4	33.20
valRS	3675	32156.5	8.75	1625	28900.7	17.78
ileRS	3945	23078.4	5.85	1698	20723.5	12.20
leuRS	3870	31360.8	8.10	1278	30571.2	23.92
gluRS	4860	25845.7	5.31	2864	24502.9	8.55
glnRS	3265	13962.0	4.27	1310	14129.4	10.78
metRS	1860	10548.6	5.67	843	9434.0	11.19
tyrRS	1840	9173.5	4.98	994	9792.9	9.85
trpRS	2260	2939.7	1.30	879	3383.7	3.84
serRS	3030	18529.5	6.11	1438	18586.3	12.92
thrRS	2890	20132.9	6.96	1824	20426.6	11.19
proRS	2750	14275.0	5.19	862	13473.6	15.63
hisRS	1450	7219.3	4.97	427	6981.9	16.35
aspRS	2050	20082.0	9.79	567	20693.3	36.49
asnRS	3660	14055.5	3.84	2140	15273.5	7.13
lysRS	2575	26206.4	10.17	1410	24330.0	17.25
alaRS	3260	37879.9	11.61	689	36929.9	53.59
glyRS	1660	31119.9	18.74	804	29038.0	36.11
pheRS	2280	12634.3	5.54	773	12144.5	15.71

References

1. Holler E and Calvin M (1972). Isoleucyl transfer ribonucleic acid synthetase of Escherichia coli B. Rapid kinetic investigation of the L-isoleucine-activating reaction. *Biochemistry*, 11(20), 3741-3752.
2. Airas RK (2018). Kinetic analysis of the isoleucyl-tRNA synthetase mechanism: the next reaction cycle can start before the previous one ends. *FEBS Open bio*, 8(2), 244-255.
3. Airas RK (2006). Analysis of the kinetic mechanism of arginyl-tRNA synthetase. *Biochimica et Biophysica Acta*, 1764(2), 307-319.
4. Bennett BD, Kimball EH, Gao M, Osterhout R, Van Dien SJ, & Rabinowitz JD (2009). Absolute metabolite concentrations and implied enzyme active site occupancy in Escherichia coli. *Nature chemical biology*, 5(8), 593-599.
5. Kern D, and Lapointe J (1980). The Catalytic Mechanism of Glutamyl-tRNA Synthetase of Escherichia coli: Evidence for a Two-step Aminoacylation Pathway, and Study of the Reactivity of the Intermediate Complex. *European Journal of Biochemistry*, 106(1), 137-150.
6. Pope AJ, Lapointe J, Mensah L, Benson N, Brown MJ, & Moore KJ (1998). Characterization of Isoleucyl-tRNA Synthetase from Staphylococcus aureus: I: KINETIC MECHANISM OF THE SUBSTRATE ACTIVATION REACTION STUDIED BY TRANSIENT AND STEADY-STATE TECHNIQUES. *Journal of Biological Chemistry*, 273(48), 31680-31690.
7. Guth EC, and Francklyn CS (2007). Kinetic discrimination of tRNA identity by the conserved motif 2 loop of a class II aminoacyl-tRNA synthetase. *Molecular cell*, 25(4), 531-542.
8. Neidhardt FC, Bloch PL, Pedersen S, & Reeh S (1977). Chemical measurement of steady-state levels of ten aminoacyl-transfer ribonucleic acid synthetases in Escherichia coli. *Journal of Bacteriology*, 129(1), 378-387.
9. Jakubowski H and Goldman E (1984). Quantities of individual aminoacyl-tRNA families and their turnover in Escherichia coli. *Journal of bacteriology*, 158(3), 769-776.
10. Choi H and Covert MW (2023). Whole-cell modeling of E. coli confirms that in vitro tRNA aminoacylation measurements are insufficient to support cell growth and predicts a positive feedback mechanism regulating arginine biosynthesis. *Nucleic Acids Research*, 51(12), 5911-5930.
11. Mori M, Zhang Z, Banaei-Esfahani A, Lalanne JB, Okano H, Collins BC, *et al.* (2021). From coarse to fine: the absolute Escherichia coli proteome under diverse growth conditions. *Molecular systems biology*, 17(5), e9536.
12. Valgepea K, Adamberg K, Seiman A, & Vilu R (2013). Escherichia coli achieves faster growth by increasing catalytic and translation rates of proteins. *Molecular BioSystems*, 9(9), 2344-2358.
13. Li GW, Burkhardt D, Gross C, & Weissman JS (2014). Quantifying absolute protein synthesis rates reveals principles underlying allocation of cellular resources. *Cell*, 157(3), 624-635.
14. Schmidt A, Kochanowski K, Vedelaar S, Ahrne E, Volkmer B, Callipo L, *et al.* (2016). The quantitative and condition-dependent Escherichia coli proteome. *Nature biotechnology*, 34(1), 104-110.
15. Peebo K, Valgepea K, Maser A, Nahku R, Adamberg K, & Vilu R (2015). Proteome reallocation in Escherichia coli with increasing specific growth rate. *Molecular BioSystems*, 11(4), 1184-1193.
16. Bremer H, and Dennis PP (2008). Modulation of chemical composition and other parameters of the cell at different exponential growth rates. *Ecosal plus*, 3(1), 10-1128.
17. Dai X, Zhu M, Warren M, Balakrishnan R, Patsalo V, Okano H, *et al.* (2016). Reduction of translating ribosomes enables Escherichia coli to maintain elongation rates during slow growth. *Nature microbiology*, 2(2), 1-9.
18. Brunschede H, Dove TL, & Bremer H (1977). Establishment of exponential growth after a nutritional shift-up in Escherichia coli B/r: accumulation of deoxyribonucleic acid, ribonucleic acid, and protein. *Journal of bacteriology*, 129(2), 1020-1033.
19. Dykeman EC (2020). A stochastic model for simulating ribosome kinetics *in vivo*. *PLoS Comp Biol*, 16(2), e1007618.
20. Dykeman EC (2023). Modelling ribosome kinetics and translational control on dynamic mRNA. *PLoS Comp Biol*, 19(1), e1010870.
21. Zhang CM, Perona JJ, Ryu K, Francklyn C, & Hou YM (2006). Distinct kinetic mechanisms of the two classes of aminoacyl-tRNA synthetases. *Journal of molecular biology*, 361(2), 300-311.
22. Ehrenberg M and Kurland CG (1984). Costs of accuracy determined by a maximal growth rate constraint. *Quarterly reviews of biophysics*, 17(1), 45-82.
23. Berg OG and Kurland CG (1997). Growth rate-optimised tRNA abundance and codon usage. *Journal of molecular biology*, 270(4), 544-550.
24. Dong H, Nilsson L, & Kurland CG (1996). Co-variation of trna abundance and codon usage in Escherichia coli at different growth rates. *Journal of molecular biology*, 260(5), 649-663.
25. Volkmer B and Heinemann M (2011). Condition-dependent cell volume and concentration of Escherichia coli to facilitate data conversion for systems biology modeling. *PloS one*, 6(7), e23126.
26. Basan M, Zhu M, Dai X, Warren M, Sevin D, Wang YP, & Hwa T (2015). Inflating bacterial cells by increased protein synthesis. *Molecular systems biology*, 11(10), 836.
27. Taheri-Araghi S, Bradde S, Sauls JT, Hill NS, Levin PA, Paulsson J, *et al.* (2015). Cell-size control and homeostasis in bacteria. *Current biology*, 25(3), 385-391.
28. Si F, Li D, Cox SE, Sauls JT, Azizi O, Sou C, *et al.* (2017). Invariance of initiation mass and predictability of cell size in Escherichia coli. *Current Biology*, 27(9), 1278-1287.
29. Belliveau NM, Chure G, Hueschen CL, Garcia HG, Kondev J, Fisher DS, *et al.* (2021). Fundamental limits on the rate of bacterial growth and their influence on proteomic composition. *Cell Systems*, 12(9), 924-944.
30. Kukko E and Heinonen J (1982). The intracellular concentration of pyrophosphate in the batch culture of Escherichia coli. *European Journal of Biochemistry*, 127(2), 347-349.
31. Kukko-Kalske E, Lintunen M, Inen MK, Lahti R, & Heinonen J (1989). Intracellular PPi concentration is not directly dependent on amount of inorganic pyrophosphatase in Escherichia coli K-12 cells. *Journal of bacteriology*, 171(8), 4498-4500.
32. Mohanty BK and Kushner SR (2019). New insights into the relationship between tRNA processing and polyadenylation in Escherichia coli. *Trends in Genetics*, 35(6), 434-445.
33. Prossliner T, Agrawal S, Heidemann DF, Sorensen MA, & Svenningsen SL (2023). tRNAs are stable after all: pitfalls in quantification of tRNA from starved Escherichia coli cultures exposed by validation of RNA purification methods. *Mbio*, 14(1), e02805-22.
34. Avcilar-Kucukgoze I, Bartholomaus A, Cordero Varela JA, Kaml RFX, Neubauer P, Budisa, N, & Ignatova Z (2016). Discharging tRNAs: a tug of war between translation and detoxification in Escherichia coli. *Nucleic acids research*, 44(17), 8324-8334.
